# Applied diagnostics in liver cancer. Efficient combinations of sorafenib with targeted inhibitors blocking AKT/mTOR

**DOI:** 10.18632/oncotarget.25766

**Published:** 2018-07-20

**Authors:** Susana Llerena, Nuria García-Díaz, Soraya Curiel-Olmo, Antonio Agraz-Doblas, Agustín García-Blanco, Helena Pisonero, María Varela, Miguel Santibáñez, Carmen Almaraz, Laura Cereceda, Nerea Martínez, María Teresa Arias-Loste, Ángela Puente, Luis Martín-Ramos, Carlos Rodríguez de Lope, Federico Castillo-Suescun, Carmen Cagigas-Fernandez, Pablo Isidro, Carlos Lopez-López, Marcos Lopez-Hoyos, Javier Llorca, Jesús Agüero, Benedicto Crespo-Facorro, Ignacio Varela, Miguel Ángel Piris, Javier Crespo, José Pedro Vaqué

**Affiliations:** ^1^ Gastroenterology and Hepatology Unit, Hospital Universitario Marqués de Valdecilla, Santander, Spain; ^2^ Infection, Immunity and Digestive Pathology Group, IDIVAL, Santander, Spain; ^3^ Translational Hematopathology Group, IDIVAL, Instituto de Investigación Marqués de Valdecilla, Santander, Spain; ^4^ Departamento de Biología Molecular, Universidad de Cantabria (UC-IBBTEC), Santander, Spain; ^5^ Josep Carreras Leukemia Research Institute and School of Medicine, University of Barcelona, Barcelona, Spain; ^6^ Digestive Service, Hepatology Unit, Hospital Universitario Central de Asturias, Oviedo, Spain; ^7^ Universidad de Cantabria-IDIVAL, Santander, Spain; ^8^ General and Digestive Tract Surgery Service, Hospital Universitario Marqués de Valdecilla, Santander, Spain; ^9^ Biobanco-Hospital Universitario Central de Asturias, Oviedo, Spain; ^10^ Oncology Service, Hospital Universitario Marqués de Valdecilla, Santander, Spain; ^11^ Immunology Service, Hospital Universitario Marqués de Valdecilla, Santander, Spain; ^12^ Department of Epidemiology and Computational Biology, School of Medicine, University of Cantabria, Santander, Spain; ^13^ CIBER Epidemiología y Salud Pública (CIBERESP), Madrid, Spain; ^14^ Microbiology Service, University Hospital Marques de Valdecilla-IDIVAL, Santander, Spain; ^15^ Department of Psychiatry, Marqués de Valdecilla University Hospital-IDIVAL, Santander, Spain; ^16^ CIBERSAM, Centro de Investigación Biomédica en Red Salud Mental, Madrid, Spain; ^17^ Department of Pathology, Fundación Jiménez Díaz, Madrid, Spain

**Keywords:** hepatocellular carcinoma, mutations, sorafenib, targeted therapy, AKT/mTOR

## Abstract

Hepatocellular carcinoma (HCC) is the third most common cause of cancer-related deaths worldwide. There is increasing interest in developing specific markers to serve as predictors of response to sorafenib and to guide targeted therapy. Using a sequencing platform designed to study somatic mutations in a selection of 112 genes (HepatoExome), we aimed to characterize lesions from HCC patients and cell lines, and to use the data to study the biological and mechanistic effects of case-specific targeted therapies used alone or in combination with sorafenib. We characterized 331 HCC cases in silico and 32 paired samples obtained prospectively from primary tumors of HCC patients. Each case was analyzed in a time compatible with the requirements of the clinic (within 15 days). In 53% of the discovery cohort cases, we detected unique mutational signatures, with up to 34% of them carrying mutated genes with the potential to guide therapy. In a panel of HCC cell lines, each characterized by a specific mutational signature, sorafenib elicited heterogeneous mechanistic and biological responses, whereas targeted therapy provoked the robust inhibition of cell proliferation and DNA synthesis along with the blockage of AKT/mTOR signaling. The combination of sorafenib with targeted therapies exhibited synergistic anti-HCC biological activity concomitantly with highly effective inhibition of MAPK and AKT/mTOR signaling. Thus, somatic mutations may lead to identify case-specific mechanisms of disease in HCC lesions arising from multiple etiologies. Moreover, targeted therapies guided by molecular characterization, used alone or in combination with sorafenib, can effectively block important HCC disease mechanisms.

## INTRODUCTION

Hepatocellular carcinoma (HCC hereafter) is the fifth most prevalent cancer and the third most frequent cause of cancer-related death worldwide, with up to 800K deaths in 2012 [1]. It is a disease of increasing incidence and the leading cause of death among patients with cirrhosis. It can be related to multiple etiologies, including infections with hepatitis B or C viruses (HBV and HCV, respectively), alcohol and nonalcoholic steatohepatitis [2, 3]. HCC diagnosis is mainly guided by radiological criteria with only one third of patients being diagnosed at early stages (namely BCLC-0 and BCLC-A) [4]. This makes them candidates for liver transplantation, surgical resection or percutaneous ablation, which is associated with a 5-year recurrence rate of 70-80% [5, 6]. Outcomes are even worse for patients with intermediate or advanced stages (BCLC-B and C, respectively) [7]. Generally, these patients will receive specific therapy that includes transarterial chemoembolization (for BCLC-B patients), which yields an increase in median survival from 16 to 24 months [8], or therapy with sorafenib (for BCLC-C patients). Sorafenib is an oral multitarget kinase inhibitor that can increase median survival from 7.9 to 10.7 months [9]. The modest but significant clinical benefit from sorafenib has prompted further clinical trials based on the comparison of sorafenib with other inhibitors, alone or in combination, as first- and second-line treatment, but these have yielded poor results [10], [11]. It is important to note that we currently lack molecular evidence to optimize the clinical benefits that HCC patients may gain from any of these therapies.

From a genomic perspective, HCC is a very heterogeneous disease, possibly reflecting the multiple etiologies causing this type of cancer [12]. Much effort has been made to characterize HCC molecularly. On one hand, whole-transcriptome analyses have revealed deregulated expression of signaling molecules, such as the overexpression of well-known oncogenic genes and pathways like MET (in 40-50% of patients) [13], IGF2 (in 10%) [14, 15], WNT/β-catenin (in 25%) [16] and TGF-β [17]. These transcriptome findings helped establish a molecular classification of two different HCC subtypes: 1) a proliferation class, with activated signaling pathways like TGF-β, MYC or PI3K-AKT, promoting worse clinical outcomes; and 2) a non-proliferation class, displaying activated WNT signaling in up to 25% of cases [17]. On the other hand, recent next-generation sequencing (NGS) mutational studies have confirmed the heterogeneous nature of HCC. The main genes recurrently found to have mutations are tumor suppressors like *TP53*, which affects 20-24% of the patients analyzed [12, 18], and those involved in the WNT pathway, like *CTNNB1*, which is detected in 33-37% of cases, or *AXIN1* (in 11-15% of cases) [11, 12, 18]. Somatic mutations have also been found in genes like *ARID1A* (in 13-17% of patients) and *CDKN2A* (7-9%), and to a lesser extent in *IRF2* (5%), *KRAS* (1.6%) and *PIK3CA* (1.6%) [12, 18]. Finally, mutations affecting the *TERT* promoter associated with increased TERT expression have been described as an early event in HCC (60% of cases) [19]. However, our knowledge of the molecular mechanisms that can participate in the development of HCC has not so far improved our ability to diagnose or treat this disease.

Taking advantage of the NGS data already generated for HCC, in this study we aimed to characterize HCC lesions to potentially use the data for diagnosis and targeted therapy. To this end, we have designed a targeted approach based on the mutational analysis of a specific selection of 112 genes, which enabled us to prospectively characterize HCC cases from patients with multiple etiologies and in a time that was compatible with the requirements of the clinic (within 15 days). Moreover, we used the data to study the biological and mechanistic effects of case-specific therapies used alone or in combination with sorafenib in a panel of HCC cell lines. This approach can enable the generation of genomic data in early stage HCCs that could be useful for tracking disease evolution and progression, and that might serve as a rationale for targeted therapy.

## RESULTS

### A targeted approach to characterizing specific mutational HCC signatures *in silico*

**Figure 1:**
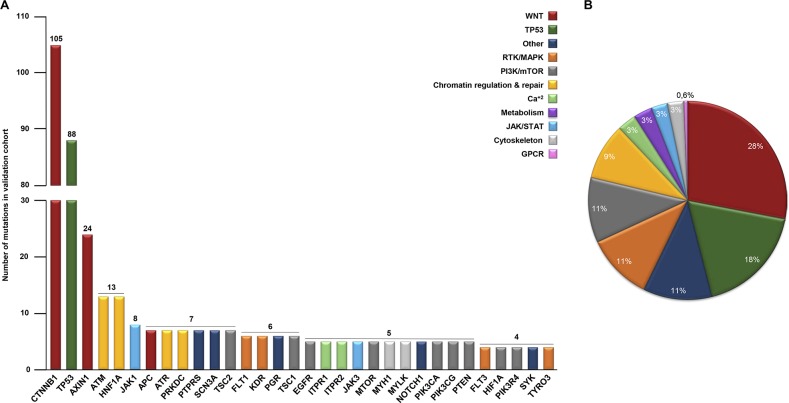
Molecular heterogeneity detected *in silico* in 331 HCC lesions with a known mutational profile **(A)** *In silico* analysis showing the number of mutations (≥ 4) detected in 331 patients (validation cohorts). **(B)** Percentage of total hits involved in the indicated signaling pathways.

There is great interest in developing novel approaches for HCC diagnosis as well as in improving our ability to manage patients with advanced diseases. We hypothesized that case-specific mutational signatures within HCC cases could act as markers of important oncogenic mechanisms involved in HCC activities, including responses to sorafenib. To explore such specific mechanisms, we designed a targeted NGS approach that focused on mutations affecting the exonic regions of a selection of 112 genes (HepatoExome hereafter). For this purpose, we used the mutational data already available from a cohort of 41 cases comprising samples from patients and cell lines (see Supplementary Table 1). Within our selection of genes, we included those already known to play a potential role in the disease (i.e., *WNT*, *B-CATENIN* and *TP53*) and others that have been implicated in specific signaling networks and that might serve as targets for therapy, e.g., JAK-STAT, PI3K-mTOR, MAPK and Receptors with Tyrosine Kinase Activity (RTKs). To assess the feasibility of our approach to detecting mutated genes in HCC samples, we studied *in silico* the mutations in genes included in the HepatoExome in an independent cohort of 331 samples from HCC patients with a known mutational profile (validation cohort in Supplementary Table 1). In this setting, we were able to detect relevant mutated genes in 69.2% of the cases. The most frequently mutated genes detected in the validation cohort samples are described in Figure 1A and Supplementary Table 2. Amongst these, we detected mutations affecting the WNT pathway (*CTNNB1*, *AXIN1* and *APC*), PI3K-mTOR (*TSC2, TSC1, PTEN* and *MTOR*), RTKs (e.g., *FLT1, EGFR, INSR* and *RET*), chromatin regulation and repair (*HNF1A, ATM, ATR* and *PRKDC*) and TP53. Interestingly, these hits belonged to multiple signaling pathways (Figure 1B), which may reflect the molecular heterogeneity associated with this disease.

### Prospective mutational profiling of HCC cases in the discovery cohort

Next, we examined the translational application of this approach by prospectively studying a cohort of 32 HCC cases arising from multiple etiologies (discovery cohort). The clinical characteristics of these 32 patients are summarized in Supplementary Table 3. The majority were male (29/32; 90.6%), and the average age of the patients was 63.8 years. All patients developed HCC in a cirrhotic liver caused by various etiologies: alcohol (12/32; 37.5%), hepatitis C virus (11/32; 34.4%), hepatitis B virus (3/32; 9.4%), hemochromatosis (3/32; 9.4%), hepatitis C virus + alcohol (2/32; 6.25%), and hepatitis B virus + alcohol (1/32; 3.2%). The samples were collected consecutively at initial stages, mostly from resection (28/32, 87.5%) but also from transplantation specimens (4/32, 12.5%). To detect somatic mutations in the HepatoExome, we compared the mutational data obtained from tumoral lesions with that from non-tumoral lesions (cirrhotic liver and blood when available). To this end, genomic DNA was extracted from paired samples from each patient and analyzed using a targeted primary ultrasequencing approach, followed by a secondary validation analysis (see supplementary methods for further details). These processes were completed within 15 days of sample reception. Interestingly, we detected somatic mutations in 17 of the 32 patients analyzed (53.1%); they had an average of 2.1 mutated genes each (Table 1).

**Table 1:** Validated somatic mutations found in the discovery cohort using HepatoExome

Patient	Chr.	Position	Ref.	Alt.	AA change	Gene	Coverage	Etiology	Associated therapy
**P-01**	2	39605221	A	T	I47K	**MAP4K3**	675	Alcohol	N/A
	3	12627258	A	C	D486E	**RAF1**	576		Selumetinib/Sorafenib
	17	7574003	G	A	R210^*^	**TP53**	704		N/A
**P-02**	3	41266110	A	C	H36P	**CTNNB1**	230	Alcohol	N/A
**P-05**	3	12660100	G	A	R41W	**RAF1**	935	Alcohol	Selumetinib/Sorafenib
	3	41266100	T	G	S33A	**CTNNB1**	325		N/A
	11	100962605	G	T	Q434K	**PGR**	206		Mifepristona
	12	26749892	G	T	T1393N	**ITPR2**	502		Tacrolimus/Cyclosporine
	22	36696277	C	A	A958S	**MYH9**	521		N/A
**P-10**	12	26568307	A	G	I2412T	**ITPR2**	45	Alcohol	Tacrolimus/Cyclosporine
**P-11**	11	532737	A	C	Y157D	**HRAS**	1384	Alcohol	Selumetinib/Sorafenib
**P-13**	1	11199401	G	A	T1697I	**MTOR**	387	HBV	Everolimus
**P-14**	14	105236685	G	A	T479M	**AKT1**	325	HBV	Everolimus/Ipatasertib
**P-16**	12	26553126	C	A	V2489L	**ITPR2**	19		Tacrolimus/Cyclosporine
**P-17**	11	108117799	G	A	R337H	**ATM**	27	HCV	N/A
	11	111625284	T	C	E196G	**PPP2R1B**	880		N/A
	17	7578370	C	A	Splice	**TP53**	743		N/A
**P-18**	3	4709191	T	C	Y600T	**ITPR1**	83	HCV	Tacrolimus/Cyclosporine
	5	38962438	T	C	Y565C	**RICTOR**	251		Everolimus
	10	43610119	C	A	G691S	**RET**	697		Regorafenib
	17	7577535	C	T	R117K	**TP53**	666		N/A
	20	54961541	A	T	F311	**AURKA**	439		Barasertib
**P-21**	3	41266124	A	G	T41A	**CTNNB1**	270	HCV	N/A
**P-22**	2	165997273	G	C	P636R	**SCN3A**	270	HCV	Zonisamida
	2	165997274	G	T	P636T	**SCN3A**	270		Zonisamida
	3	41266137	C	T	Y157D	**CTNNB1**	161		N/A
**P-23**	3	41266110	A	C	H36P	**CTNNB1**	161	HCV	N/A
**P-25**	3	41268766	A	C	K335T	**CTNNB1**	108	HCV	N/A
**P-26**	3	41266113	C	A	S37Y	**CTNNB1**	87	HCV	N/A
	4	55981463	G	T	N158K	**KDR**	130		Sorafenib
**P-31**	3	41266136	T	C	S45P	**CTNNB1**	343	Hemochr.	N/A
	6	44219910	A	T	D546V	**HSP90AB1**	934		N/A
**P-32**	2	165948799	A	G	I1542T	**SCN3A**	928	Hemochr.	Zonisamida
	3	41266101	C	G	S33C	**CTNNB1**	51		N/A
	12	26636635	T	C	N2003S	**ITPR2**	278		Tacrolimus/Cyclosporine
	17	7577094	G	A	R150W	**TP53**	452		N/A

Patient: Patient number; Chr.: Chromosome number; Position: Genomic location of the mutation in the chromosome; Ref.: Normal nucleotide; Alt.: Altered nucleotide; AA change: Amino acid change; Gene: Gene name; Coverage: Number of reads analyzed at each position; Etiology: Etiology of each patient; Associated therapy: Possibly Associated Therapy for the indicated genes and/or signaling pathways. Hemocrom.: Hemochromatosis.

Moreover, each patient showed a unique mutational profile with individualized combinations of mutations and mutated genes. Considered in greater detail, our results identified mutant genes that can participate in a number of signaling pathways, as would be expected from our previous *in silico* observations. Among these, we found mutations affecting WNT-β-CATENIN signaling (*CTNNB1* in 8/32 of the samples), the MAPK pathway (*RAF1* and *HRAS*; 3/32 samples), intracellular calcium signaling in 5/32 of the samples (*ITPR1* and *ITPR2*), and members of the PI3K/mTOR pathway (*MTOR, AKT1* and *RICTOR*; 3/32 samples) (Table 1). Somatic mutations were detected with average depths of 420-X and 3.25K-X in the primary and validation analyses, respectively.

Thus, it is possible to use this approach to characterize cancer lesions in up to 53% of patients with HCC with respect to the presence of genomic alterations that presumably affect specific signaling mechanisms.

### Heterogeneous mechanistic effects on treatment with sorafenib in HCC cells

**Figure 2:**
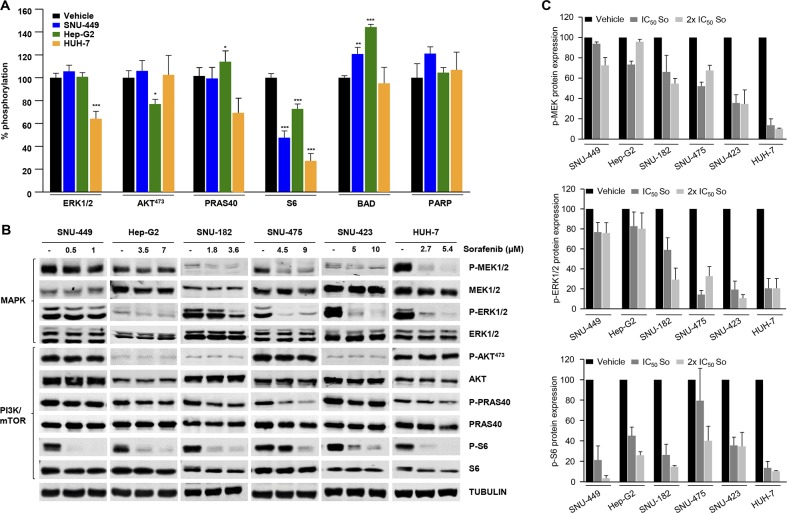
Mechanistic effects of sorafenib in a panel of HCC cell lines **(A)** Intracellular signaling array of SNU-449, Hep-G2 and HUH-7 cells starved and treated for 1h with their IC_50_ concentration of Sorafenib. **(B)** Western blotting analyses of SNU-449, Hep-G2, SNU-182, SNU-475, SNU-423 and HUH-7 cells starved and treated for 1h with control vehicle (−) and the IC_50_ and 2x IC_50_ concentrations of sorafenib, as indicated. Cell lysates were incubated with P-MEK1/2, MEK1/2, P-ERK1/2, ERK1/2, P-AKT473, AKT, P-PRAS40, PRAS40, P-S6, S6, and α-tubulin antibodies. **(C)** P-MEK, P-ERK1/2 and P-S6 relative to MEK, ERK1/2 and S6 protein expression in HCC cell lines treated with control vehicle, the IC50, and 2 x IC_50_ concentrations of sorafenib. Error bars show SEM. ^*^ compared with the control vehicle (^*^ P < 0.05; ^**^P < 0.01; ^***^P < 0.001).

Sorafenib is the only inhibitor used to treat HCC at advanced stages in the clinic. We sought to explore the effects in proliferation that treatment with this inhibitor could elicit in a panel of HCC cell lines. We first performed an *in silico* characterization that enabled the detection of mutations in the genes included in the HepatoExome. As expected from our previous observations in lesions from HCC patients, each cell line showed an individual and unique mutational profile (Supplementary Table 4). In this setting, the IC_50_ of sorafenib differed between cell lines over a range between 0.5 and 5 μM (Supplementary Figure 1 and Supplementary Table 4). This observation led us to compare the mechanistic effects of sorafenib on the activity of some well-known intracellular cancer-related signaling pathways using an intracellular pathway array kit (see Methods). To this end, we incubated SNU-449, Hep-G2 and HUH-7 cells with their specific IC_50_ concentrations of sorafenib. Under these conditions, most of the pathways showed no response to this drug, e.g., JAK/STAT, JNK, p53, members of the PI3K/mTOR pathways (such as AKT, mTOR and PRAS40) or the proapoptotic CASPASE-3 and PARP (with the exception of P-BAD in Hep-G2 cells) (Figure 2A and Supplementary Table 5). On the other hand, treatment with sorafenib in HCC cells elicited heterogeneous inhibitions of ERK1/2 (MAPK) and RPS6 (S6) phosphorylation (PI3K/mTOR) alongside a consistent activation of P-AMPK between cell lines (Supplementary Table 5). Since MAPK and PI3K/mTOR are well known signaling pathways downstream of the intended molecular targets of sorafenib, we sought to confirm these data by using an independent approach in a larger panel of HCC cells lines. To this end, we performed western blot (WB) using lysates from starved cells treated with 1- and 2-fold IC_50_ concentrations of the drug. Confirming our previous results, sorafenib displayed differential abilities to inhibit the P-MEK and P-ERK signaling pathway in different cell lines, independently of the drug concentration (Figure 2B and 2C). In this regard, the effects of this inhibitor on MEK-ERK activity ranged from no inhibition (SNU-449 cells) through medium (SNU-182) to high (HUH-7) levels of inhibition. Finally, under these conditions, sorafenib inhibited P-S6 at different intensities (compare the IC_50_ responses in SNU-475, Hep-G2 and HUH-7 cells) and had no effect on P-AKT (Figure 2B and 2C). Thus, in the context of a variety of HCC cell lines, sorafenib elicited heterogeneous proliferative and mechanistic responses downstream of its intended molecular targets.

### *Ex vivo* effects of targeted therapies guided by individual mutational profiles

**Figure 3:**
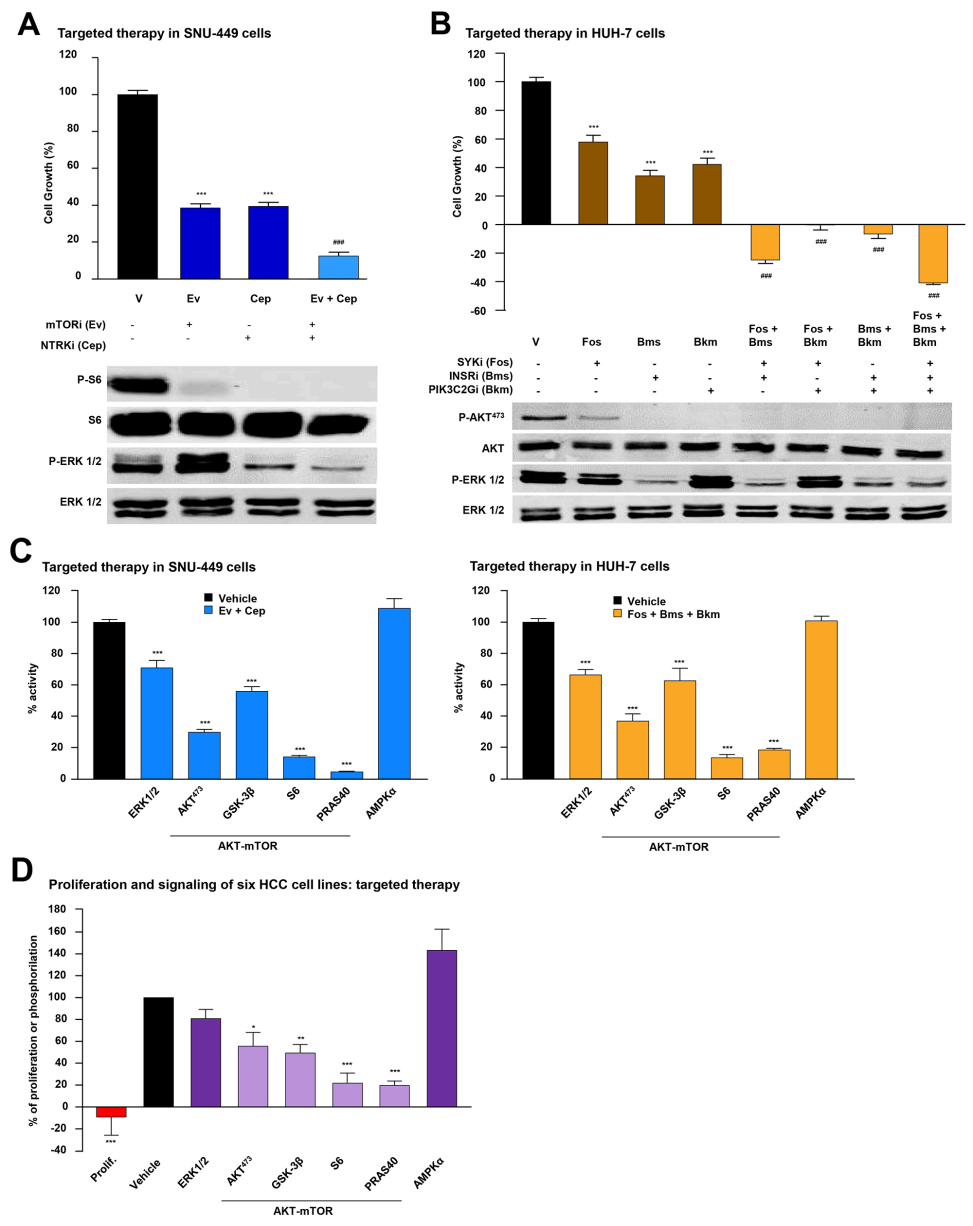
Biological effects of combined targeted therapies in HCC cells **(A)** *Top*: Proliferation analysis of SNU-449 cells incubated 48h with control vehicle (V, black bar) or the IC_50_ concentration of the indicated inhibitor (mTORi (Ev: Everolimus) and NTRKi (Cep: Lestaurtinib)) alone (dark blue bars) or in a double (light blue bar) combination. *Bottom*: Western Blotting analyses of SNU-449 cells starved and treated for 1h with control vehicle or the indicated inhibitor, or the combination of inhibitors under the same conditions as above, and incubated with P-S6, S6, P-ERK1/2 and ERK1/2 antibodies. **(B)** *Top:* Proliferation analysis of HUH-7 cells at 48h incubated with control vehicle (V, black bar) or the IC_50_ concentration of the indicated inhibitor (SYKi (Fos: Fostamatinib), INSRi (Bms: BMS-754807) and PIK3R2i (Bkm: Buparlisib)) alone (dark brown bars), or in double or triple combination (light brown bars). *Bottom:* Western Blotting analysis of HUH-7 cells treated for 1h with control vehicle, the indicated inhibitor, or the combination of inhibitors under the same conditions as above. P-AKT^473^, AKT, P-ERK1/2 and ERK1/2, antibodies were used as indicated. **(C)** Intracellular signaling array of SNU-449 (*left*) and HUH-7 (*right*) cells starved and treated for 1h with control vehicle (black bar) or the combination of IC_50_ concentration of the indicated targeted inhibitors. **(D)** Proliferation (red bar) and phosphorylation of the indicated antibodies (purple bars) within a panel of six HCC cell lines compared to control vehicle (black bar). Error bars show SEM. ^*^ compared with the control vehicle (^*^ P < 0.05; ^**^P < 0.01; ^***^P < 0.001). ^#^ compared with each inhibitor alone (^###^ P < 0.001).

To gain insights into the biological and mechanistic effects that targeted therapy guided by mutational profiles could exert in HCC cells, we decided to focus on the potentially actionable mutations in our panel of human HCC cell lines. Using SNU-449 and HUH-7 cells as examples of low or high MAPK inhibition by sorafenib, respectively, we first detected mutations in specific genes (and validated them by Sanger sequencing; Supplementary Figure 2 and Supplementary Table 6). For SNU-449 cells, the mutated genes were *NTRK1* and *PTEN*, which were associated with the inhibitors lestaurtinib (Cep) and everolimus (Ev) respectively (Table 2). In HUH-7 cells, mutated genes like *INSR*, *SYK* and *PIK3C2G* were associated with fostamatinib (Fos), BMS-754807 (Bms) and buparlisib (Bkm) respectively, (Table 2). We then analyzed the anti-proliferative effects of each inhibitor, in the intended cell line, and calculated their IC_50_ concentrations, using them in the subsequent experiments (Table 2). In addition to the biological effects observed with targeted drugs, incubation of starved cells with 1- or 2-fold IC_50_ concentrations of each drug inhibited downstream signaling pathways associated with the activity of the mutated genes (Supplementary Figure 3). The biological and mechanistic effects of the drugs, used alone or in combinations, were then compared in SNU-449 and HUH-7 HCC cells. In these settings, all combinations were highly effective at inhibiting cell proliferation compared with single treatments, suggesting that multiple mechanisms associated with case-specific mutations could participate in controlling essential HCC activities (Figure 3A and 3B). Furthermore, similar results were also obtained in other HCC cell lines like Hep-G2 (Table 2 and Supplementary Figure 4), SNU-182, SNU-475 and SNU-423 (Table 2 and Supplementary Figure 5). Following the example using SNU-449 and HUH-7 cells, we comparatively studied the molecular effects elicited by case-specific combinations of targeted therapy over multiple signaling pathways simultaneously. Interestingly, we only found significant inhibition over the activities of MAPK-ERK and a number of AKT/mTOR pathway effectors like AKT^473^, GSK-3B, S6 and PRAS40 (Figure 3C and Supplementary Table 5). These results were further confirmed alongside our panel of six different cell lines, each treated with a specific targeted therapy defined by their individual mutational signatures (Table 2). In these settings, targeted therapy provoked a robust inhibition of cell proliferation that occurred alongside a highly significant inhibition of PRAS40 and S6 activities, hence suggesting an important role for these molecules, which are downstream effectors of the AKT/mTOR pathway, in the biology of HCC (Figure 3D and Supplementary Table 7).

**Table 2:** Potentially actionable mutations found in silico in HCC cell lines and IC50 values associated to them. Table showing the mutational characteristics of six commercial cell lines (in silico comparison with Cancer Cell Line Encyclopedia (CCLE) data)

Cell line	Chr.	Position	Ref.	Alt.	AA change	Gene	Inhibitor name	Inhibitor	IC_50_ (μM)
**Hep-G2**	4	55976843	A	T	Y357N	KDR	KDRi (So)	SORAFENIB	3,5
	10	43608351	G	A	D567N	RET	RETi (Re)	REGORAFENIB	3,1
	19	18279692	C	A	Y655^*^	PIK3R2	PIK3R2i (Bkm)	BUPARLISIB (BKM-120)	2,8
**SNU-449**	1	156851421	A	C	D793A	NTRK1	NTRKi (Cep)	LESTAURTINIB (CEP-701)	1,6
	10	89717696	T	C	F241L	PTEN	mTORi (Ev)	EVEROLIMUS	14,6
**HUH-7**	9	93606577	A	G	K133E	SYK	SYKi (Fos)	FOSTAMATINIB	12,9
	12	18762561	A	C	I1394L	PIK3C2G	PIK3C2Gi (Bkm)	BUPARLISIB (BKM-120)	1,7
	19	7141798	T	C	T858A	INSR	INSRi (Bms)	BMS-754807	8,5
**SNU-182**	3	130409498	T	A	R1033S	PIK3R4	PIK3R4i (Bkm)	BUPARLISIB (BKM-120)	0,8
	13	29008268	T	A	E201D	FLT1	FLT1i (Re)	REGORAFENIB	4,7
	19	7184495	G	A	P269L	INSR	INSRi (Bms)	BMS-754807	1,5
**SNU-475**	13	28611336	G	A	T432M	FLT3	FLT3i (Cep)	LESTAURTINIB (CEP-701)	2,5
**SNU-423**	3	130452809	C	A	V345F	PIK3R4	PIK3R4i (Bkm)	BUPARLISIB (BKM-120)	1,7
	13	28913428	C	T	E789K	FLT1	FLT1i (Re)	REGORAFENIB	9,5

The table includes Cell line: Cell line name; Chr.: Chromosome number; Position: Genomic location of the mutation in the chromosome; Ref.: Normal nucleotide; Alt.: Altered nucleotide; AA Change: Amino acid change; Gene: Gene name; Inhibitor name: Used throughout this report; Inhibitor: General name and IC50 (μM): Micromolar IC50 concentration for the indicated inhibitor.

### Biological and mechanistic effects of combinations of sorafenib with targeted therapies in HCC cell lines

**Figure 4:**
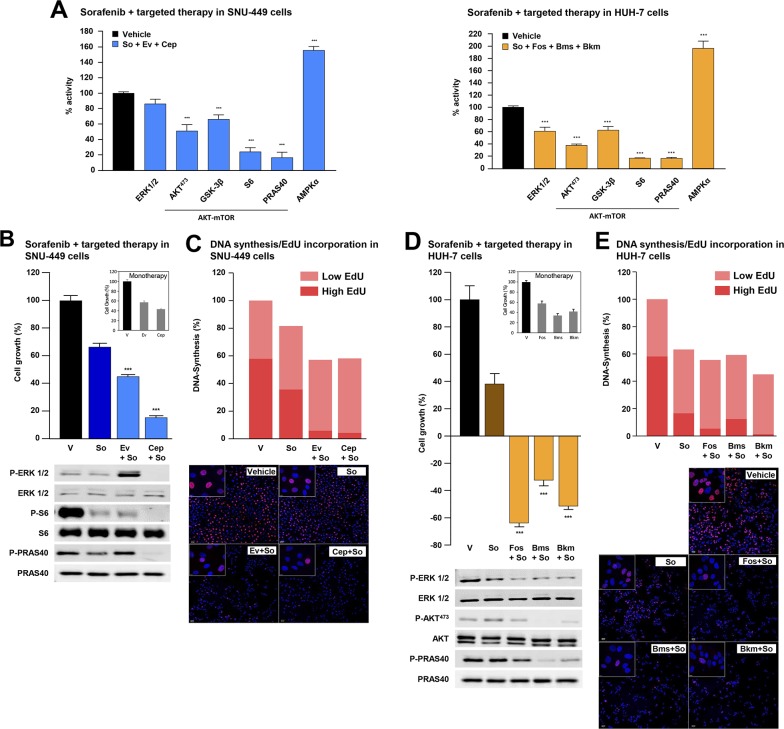
Combination of sorafenib and targeted therapy in HCC cells **(A)** Intracellular signaling array of SNU-449 (*left*) and HUH-7 (*right*) cells starved and treated for 1h with control vehicle or the IC_50_ concentrations of the indicated inhibitors. Proliferation analyses of SNU-449 (**B**, *top*) and HUH-7 cells (**D**, *top*) at 48h incubated with control vehicle (V) or the IC_50_ concentration of sorafenib alone (dark blue and dark brown bars for SNU-449 and HUH-7 cells, respectively), the targeted inhibitors alone (inner squares) or combinations of sorafenib with targeted inhibitors (light blue and light brown bars for SNU-449 and HUH-7 cells, respectively). Western Blotting analyses of SNU-449 (B, *bottom*) and HUH-7 (D, *bottom*) cells starved, treated under the same conditions as above and incubated with P-ERK1/2, ERK1/2, P-S6, S6, P-PRAS40, PRAS40, P-AKT^473^ and AKT antibodies. DNA synthesis assay using Click-iT® EdU in SNU-449 **(C)** and HUH-7 **(E)** cells incubated for 24h under the same conditions as in B or D respectively. Graph bars show percentage of low (light red) or high (intense red) EdU-stained cells in three photographic fields from a representative experiment. Representative pictures show the nucleus of the total number of cells (blue dots) and EdU-positive cells (red dots). Statistical analyses of targeted therapy or sorafenib plus targeted therapy versus sorafenib alone. Error bars show the SEM. ^*^ P ≤ 0.05; ^**^P ≤ 0.01; ^***^P ≤ 0.001.

In light of our findings, it is conceivable that case-specific targeted therapies could increase the anti-HCC effects of sorafenib. To explore this possibility, we compared the mechanistic effects elicited by incubating SNU-449 and HUH-7 cells with IC_50_ concentrations of sorafenib plus cell-specific targeted therapies (see results from Hep-G2 cells in Supplementary Figure 4). As expected from our previous findings, these combinations most strongly inhibited specific signaling mechanisms like MAPK-ERK (P-ERK-1/2) and AKT/mTOR (P-AKT^473^, P-GSK-3B and P-PRAS40 and P-S6), which are known to play an important role in the biology of HCC (Figure 4A and Supplementary Table 5). We next examined our data by using an alternative approach to explore the biological and mechanistic effects of treatment with sorafenib and targeted therapy used alone or in combination in SNU-449 and HUH-7 cells. The combination of sorafenib with targeted drugs most strongly inhibited cell proliferation and DNA synthesis in HCC cells (Figures 4B-4E). Interestingly, the combination of sorafenib with targeted inhibitors caused synergistic effects over the proliferation of SNU-449 and HUH-7 cells, with combination indexes below 1 (Supplementary Figure 6). Moreover, this occurred in parallel with higher blockages of MAPK and AKT/mTOR signaling pathways as assessed by western blot using anti-P-ERK-1/2, anti-P-AKT^473^ and anti-P-PRAS40 antibodies (Figures 4B-4E). Similar results were also obtained in other HCC cell lines like Hep-G2, SNU-182, SNU-475 and SNU-423 (Supplementary Figures 4 and 5). Finally, we interchanged the targeted inhibitors plus sorafenib between SNU-449 and HUH-7 cells and analyzed the effects in cell proliferation. Our data show higher efficiency when inhibitors were used in the appropriate mutational background (compare Supplementary Figures 7A with 4D and Supplementary Figure 7B with 4B). It is therefore possible that, using our targeted approach, we could detect and target specific mechanisms, like for example AKT/mTOR, that when used in combination with sorafenib, could increase its anti-HCC activities.

## DISCUSSION

Considerable effort has been made to determine the main mechanisms that may be involved in the pathogenesis and evolution of HCC. Based on the NGS data already available in the literature concerning HCC, we designed a targeted approach to characterize HCC cases from patients with multiple etiologies and in a time compatible with the requirements of clinics (within 15 days). This method enables us to show that sorafenib can produce heterogeneous cellular responses in different genomic contexts, and that case-specific targeted inhibitors can greatly increase its biological and mechanistic anti-HCC effects.

Taking advantage of the genetically heterogeneous nature of HCC, we set up a genomic platform consisting of an HCC customized HaloPlex^TM^ enrichment library coupled to a MiSeq sequencing system. This enabled us to characterize HCC cases (in initial tumors) for the presence of somatic mutations in a selection of 112 genes cost-effectively and in suitably quick manner to meet the requirements of the clinic. We first performed an *in silico* analysis of 331 HCC cases (validation cohort) and a prospective *ex vivo* study of a cohort of 32 paired samples (discovery cohort) from multiple etiologies and low-stage HCC. Independently of the etiology of the tumor, 60% of all cases displayed unique mutational profiles; up to 34% of the discovery cohort cases had mutated genes that could be associated with an inhibitor. In this regard, *RAF1* (patient-01; sorafenib or selumetinib), *MTOR* (patient-13; everolimus) and *RET* (patient-18; regorafenib) are but three examples of mutated genes potentially associated with specific therapy. We might be able to use the information obtained by this method to track disease progression, for example, using liquid biopsies, and to design case-specific approaches for therapy.

Treatment with sorafenib is the standard of care in HCC patients with advanced disease (BCLC-C) [9]. It is a multi-kinase inhibitor that can target VEGFR, PDFGR, c-Kit, c-RAF and B-RAF activities and currently offers limited clinical benefits [9, 20]. To study the molecular mechanisms targeted by sorafenib in a heterogeneous genetic context, we analyzed a panel of HCC cell lines, each of which had a unique mutational profile. Surprisingly, we found heterogeneous biological responses between the cell lines with respect to the range of IC_50_ concentrations observed. In addition, from a mechanistic perspective, treatment with sorafenib elicited inhibitory responses of different intensities to MAPK-ERK and PI3K/mTOR activities depending on the cell line tested. On the other hand, we observed steady P-AMPK activation, as have previously been described as potential mechanisms involved in cellular responses to this drug [21, 22]. It is possible that, in different genomic contexts, as in this case of HCC cell lines with different mutational profiles, the intracellular mechanistic effects elicited by sorafenib may vary. It is also conceivable that this could be reflected in the heterogeneous population of patients that are uniformly treated with sorafenib and develop variable and unpredictable clinical responses to this drug.

We examined the HepatoExome data obtained in a panel of HCC cell lines by analyzing the effects of targeted drugs guided by case-specific mutational profiles. We found that a rational combination of targeted inhibitors can strongly inhibit HCC cell proliferation. Moreover, case-specific combinatory therapies were highly effective at blocking important HCC signaling mechanisms like MAPK or AKT/mTOR. In this context, downstream of AKT/mTOR signaling axis, we detected two effectors like RPS6 (S6) and PRAS40, which were highly dephosphorylated and presumably inactivated in response to different targeted therapies. Phosphorylation of S6 by p70-S6K has been shown to regulate protein synthesis and promote cell growth and proliferation by selectively promoting the translation of specific mRNAs [23, 24]. PRAS40 can be directly phosphorylated by AKT and exert pro-tumorigenic activities. Interestingly, in its dephosphorylated form PRAS40 negatively regulates mTOR activity which can be reversed by direct phosphorylation (reviewed in [25]). It is thus possible, that guided by molecular characterization of tumor lesions, we could use PI3K, AKT or mTOR inhibitors to disrupt AKT/mTOR signaling and inhibit S6 and PRAS40 activities as an effective approach to treat HCC. We believe this study highlights what targeted characterization of specific lesions might offer by way of diagnostic possibilities for human hepatocarcinoma in the near future. Using early stage hepatocarcinoma samples, we found highly heterogeneous genomic landscapes with unique mutational signatures. It is conceivable that these can trigger aberrant activation of multiple mechanisms that may contribute to the pathogenesis and progression of each disease. Following this line of evidence, it is also possible that upon molecular characterization we could use this heterogeneity as a molecular basis to detect specific mechanisms promoting HCC progression and resistance to treatment and to serve as potential targets for therapy. In this regard, multiple clinical trials have been conducted to explore the clinical benefit of other drugs when compared, or used in combination, with sorafenib (reviewed in [11]). These have yielded poor results that could be partially explained by the inclusion of an uncharacterized population of patients in the studies. We also explored the effects of combinations of sorafenib with case-specific inhibitors in a panel of HCC cell lines. In each case, we observed greater inhibition of cell proliferation and DNA synthesis of the drug combinations (targeted inhibitors + sorafenib) compared with sorafenib alone. Intriguingly, these inhibitory effects were more evident in HCC cells, in which treatment with sorafenib alone inhibited MAPK-ERK signaling compared with those in which it did not (HUH-7 and SNU-449 cells, respectively; see data in Figures 2 and 4). Moreover, our results suggest that targeted blockage of MAPK-ERK signaling and AKT/mTOR used along with sorafenib can greatly inhibit cell proliferation and DNA synthesis of HCC cells. Thus, our results suggest that molecular characterization of HCC cases could help develop therapies that are more efficient. In this regard, a phase II trial of tivantinib used in the second line, showed no difference in survival compared with a placebo. This inhibitor is currently being tested as a highly selective MET inhibitor, although the exact mechanism of action is still unclear [26]. Nevertheless, a subgroup of patients with a high level of MET expression significantly benefited from this treatment although they had worse survival overall; a phase III trial in this specific population has since been designed [27].

Despite its potential applicability in routine clinical practice, our approach requires several limitations to be overcome in a similar way to those described in [28]. In the case of HCC, a solution would entail: 1) establishing efficient protocols to safely collect, manipulate and characterize specific lesions that are representative of the advanced steps of this disease; 2) considering other molecular approaches, such as transcriptome or copy number variation studies, in addition to targeted mutational analyses; 3) managing the toxicity due to drug combinations, particularly given that this disease usually appears in the context of a damaged liver; and 4) dealing with tumor heterogeneity and interactions with the immune system that may be responsible for the resistance eventually acquired after combination treatments.

In summary, adopting targeted approaches to characterize HCC lesions may make it possible to detect specific disease mechanisms, like for example AKT/mTOR, that can lead to: 1) develop biomarkers to support diagnosis and/or prognosis; 2) serve as targets for specific inhibitors rationally combined in individualized therapies to target case-specific mechanisms of hepatocyte transformation; and 3) design more effective combination therapies when used with sorafenib in advanced stages of HCC.

## MATERIALS AND METHODS

### Patient samples

Matched tumoral and non-tumoral samples from 32 patients with clinically characterized HCC who were surgically treated (resection or transplant) were obtained retrospectively and prospectively (discovery cohort; Supplementary Table 1): 17 patients from Hospital Universitario Marqués de Valdecilla (HUMV), Santander; and 15 from Hospital Universitario Central de Asturias (HUCA), Oviedo. Tumoral DNA samples were obtained from freshly frozen (FF) tissue samples and matched non-tumoral DNA was collected from FF adjacent cirrhotic tissue samples and/or peripheral blood from the available patients.

All human samples used in this study were collected following the Declaration of Helsinki protocols after obtaining written informed consent from each patient as required by the CEIC (Comité Ético de Investigación Clínica, Cantabria) and the CEAS (Comité de Ética para la Atención Sanitaria, Oviedo). No donor organs were obtained from executed prisoners or other institutionalized persons.

### Genomic DNA samples

Genomic DNA was extracted from fresh (blood) and/or frozen (cirrhotic and tumoral liver) using standard procedures. Briefly, PBS-washed samples, were centrifuged and lysed using “Tissue and cell lysis solution” buffer for the MasterPureTM kit, complemented by proteinase K (5 μl/100 μl buffer) (Epicenter), shaking overnight at 56°C. DNA was extracted using phenol/chloroform/isoamyl alcohol (in proportions of 25:24:1, respectively) in a fast Lock Gel Light Eppendorf tube (Eppendorf), then washed and precipitated. Genomic DNA was quantified using a Qubit ds DNA BR assay kit and a Qubit 2.0 fluorimeter (Invitrogen).

### Enrichment library design, preparation and sequencing

Targeted enrichment sequencing was performed on human FF tumor and non-tumor specimen and, when indicated, on blood samples. The custom probe design was constructed with SureDesign (Agilent Technologies; Design ID: 37503-1413372517). The design focused on the coding regions of a group of 112 genes known to be mutated in HCC, and which were selected based on the following criteria: i) genes of known relevance in HCC, ii) genes that may be associated with pharmacological inhibitors with potential clinical use and iii) genes shown mutated in HCC independently of the population frequency. DNA libraries were prepared with the HaloPlex Target Enrichment System, following the manufacturer's instructions and sequenced as described in [28].

### Somatic mutation identification and validation

Somatic mutation identification was done by using Agilent Sure Call 2.1.1.13 software and IGV 2.3.46 software. In parallel Sequencing data were aligned against the human reference genome (hg19) using the BWA aligner [29]. The alignment was refined using SAMTOOLS fixmate and PICARD TOOLS cleanSam tools [30], (http://broadinstitute.github.io/picard.). The RAMSES application was used to detect nucleotide substitutions [31]. For validation, genomic DNA was amplified using the specific oligonucleotides described in Supplementary Table 5. Samples were prepared and analyzed as described in [28].

### Cell cultures and reagents

Six human hepatocellular carcinoma cell lines were used. Hep-G2, SNU-449, SNU-475, SNU-423 and SNU-182 cells were obtained from the American Type Cell Collection (ATCC, Rockville, MD). HUH-7 cells were obtained from the Japanese Collection of Research Bioresources Cell Bank (JCRB, Japan). Genomic data from these cells, including the somatic mutations detected in this study, are publicly available at the Broad-Novartis Cancer Cell Line Encyclopedia website (CCLE: http://www.broadinstitute.org). Commercial cell lines were cultured as recommended by ATCC or JCRB. Hep-G2 and HUH-7 cells were cultured in EMEM medium (Lonza, Basel, Switzerland) and SNU-475, SNU-449, SNU-423 and SNU-182 cells were cultured in RPMI-1640 medium (Lonza, Basel, Switzerland). Both mediums were supplemented with 10% heat-inactivated fetal bovine serum (FBS) (Life Technologies), glucose (4.5 g/L), L-glutamine (292 mg/L), streptomycin sulfate (10 mg/L) and potassium penicillin (10000 U/L) (Lonza).

To perform functional analysis the following inhibitors used in this study were obtained from Selleck Chemicals (Houston, TX): BMS-754807, Buparlisib (BKM-120), Everolimus (RAD001), Fostamatinib (R788), Regorafenib (BAY 73-4506) and Sorafenib; and Lestaurtinib (Cep-701) inhibitor were obtained from Sigma Aldrich. These drugs were reconstituted in Dimethyl Sulfoxide (DMSO) and kept at −20°C until use.

### Statistics

Unless otherwise specified, all experiments were done in independent triplicates and all numerical data were summarized as the average of the values ± the standard error of the mean (SEM) using GraphPad Prism5 software. In Figure 3D and Supplementary Table 6, the significate effects of targeted therapy versus control were calculated for each cell line. Each global median was compared using t-Student test.

## SUPPLEMENTARY MATERIALS FIGURES AND TABLES










