# Supplementary material for: Applied diagnostics in liver cancer. Efficient combinations of sorafenib with targeted inhibitors blocking AKT/mTOR
Source: Oncotarget. 2018 Jul 20;9(56):30869–82. doi: 10.18632/oncotarget.25766 (PMC6089396; doi:10.18632/oncotarget.25766)
Supplement: Supplementary file 1 [file oncotarget-09-30869-s001.pdf]

Applied diagnostics in liver cancer. Efficient combinations of sorafenib with targeted inhibitors blocking AKT/mTOR

SUPPLEMENTARY MATERIALS

Enrichment library design, preparation and sequencing

Targeted enrichment sequencing was performed on human FF tumor and non-tumor specimen and, when indicated, on blood samples. The custom probe design was constructed with SureDesign (Agilent Technologies) enriching for the exons of 112 genes of interest (Design ID: 37503-1413372517). The design focused on the coding regions of a group of 112 genes known to be mutated in HCC, and which were selected based on the following criteria: i) genes of known relevance in HCC, ii) genes that may be associated with pharmacological inhibitors with potential clinical use and iii) genes shown mutated in HCC independently of the population frequency.

DNA libraries were prepared with the HaloPlex Target Enrichment System, following the manufacturer's instructions. Briefly, 400 ng of genomic DNA was digested with the specific cocktail of restriction enzymes provided in the kit. Digested DNA was then hybridized to a probe for target enrichment, indexed and captured. Each DNA was then amplified by PCR at Tm = 60ºC, for 18 cycles, using a Herculase II Fusion Enzyme kit (Agilent Technologies). Next, amplified target libraries were purified using an Agencourt AMPure XP Kit (Beckman Coulter Genomics), following the manufacturer's instructions, and quantified with Qubit 2.0 fluorimeter (Life Technologies), using the Qubit® dsDNA HS Assay Kit (Life Technologies). They were also analyzed in parallel by capillary electrophoresis in a 2100 Bioanalyzer (Agilent Technologies), using High Sensitivity DNA reagents and chip Kits (Agilent Technologies). Libraries were sequenced at the Instituto de Medicina Genómica (IMEGEN, Valencia University, Spain) with a MiSeq Personal Sequencer (Illumina).

Somatic mutation identification

Somatic mutation identification was done by using Agilent Sure Call 2.1.1.13 software and IGV 2.3.46 software. In parallel Sequencing data were aligned against the human reference genome (hg19) using the BWA aligner [1]. The alignment was refined using SAMTOOLS fixmate and PICARD TOOLS cleanSam tools [2], (http://broadinstitute.github.io/picard.). Local realignment of insertions and deletions (indels) was then performed using the GATK suite [2] before final sorting and indexing. The RAMSES application, written in-house, was used to detect nucleotide substitutions [3]. Small indels were identified using Pindel in paired tumor-normal mode [4]. For greater specificity, only simple insertion and deletion events of fewer than 10 bp were selected. An in-house perl script filter was used to extract high-quality indels: considering the high sequence coverage obtained in these samples, only those indels with a minimum coverage of 20 reads in both tumor and normal samples, and with a minimum frequency of 10% of the reads and a minimum of five independent reads supporting the event in the tumor sample, and with no evidence in the normal sample, were considered. All potential somatic mutations were filtered using the dbSNP132 and 1000 Genomes Project mutation databases and the functional consequence at the protein level was annotated according to the Ensembl database using an in-house perl script based on the Ensembl database API.

Validation

Genomic DNA was amplified using the specific oligonucleotides described in Supplementary Table 5. All amplicons from the same patient were mixed in a tube and each sample was quantified by Qubit 2.0 (Life Technologies), using the Qubit® dsDNA BR Assay Kit (Life Technologies). 500 ng of each DNA sample was repaired using NEBNext: Ultra End Repair/dA Tailing Module kit (Biolabs) and linked to a pair of adapters; 3'-end and 5'-end, respectively. Then, a pair of indexing primers was bound to the adapters to allow subsequent identification of each sample. DNA was purified with Agencourt AMPure XP beads (Beckman Coulter) and 4 ng of each DNA was sequenced by Next Generation Sequencing, using a MiSeq Personal platform (Illumina).

Cell viability and synergism analyses

Cells were seeded in a 96-well plate at a density of 1,000 cells (for Hep-G2, SNU-423, SNU-449 and SNU-475 cells), 2,000 cells (for HUH-7 cells) or 3,000 cells (for SNU-182 cells) per well overnight at 37ºC (with 10% CO_2 and at 96% RH) unless otherwise stated. After that time, cells were attached and exponentially grown to approximately 50% confluence, and the appropriate concentrations of inhibitors were added in each case to the medium.

Cellular proliferation was evaluated using CellTiter-Glo® Luminescent Cell Viability Assay (Promega) and luminometric changes were quantified using the Synergy™ HTX Multi-Mode Microplate Reader (Biotek).

IC_{50} dose was estimated by using increasing concentrations of each drug and analyzing those data with GraphPad Prism 5 software (GraphPad Software Inc., La Jolla, CA, USA).

CalcuSyn software (version 2.11, Biosoft, Cambridge, UK) was used to analyze and generate combination index (CI) values, as previously described [5]. This software assesses whether a combination of two drugs results in a synergistic (CI < 1), additive (CI = 1) or antagonistic effect (CI > 1). This method considers the fraction of affected cells of both monotherapies and compares this with the fraction of affected cells of the combination therapies.

DNA synthesis assays

To assess the effects on DNA synthesis, cells were grown in 12mm coverslips to approximately 70% confluence. After 24h of treatment with indicated inhibitors, cells were incubated for a further 2 h with Click-iT® EdU (Alexa Fluor® 594 Imaging Kit; Life Technologies, C10339). Immediately afterwards, cells were fixed using 3.7% formaldehyde in PBS and permeabilized with 0.5% Triton X-100. Finally, DNA was stained using Hoechst 33342 1:2000 in PBS. Cell images were captured with a Nikon A1R confocal microscope with Plan Apo 10x/0.45NA and Plan ApoVC 60x/1.40NA objectives. A 405-nm laser diode was used to excite Hoechst 33342 and a 561-nm laser diode was used to excite Alexa Fluor 594. Images were processed and analyzed using the object count tool of Nis elements software. Briefly, red and blue nuclei were segmented, channels were separated, and all images thresholded with the same parameters. Finally, the fluorescence of the segmented nuclei was measured. The median of fluorescence intensity of all images was set up as threshold. All measurements below this threshold were considered low EdU whereas all measurements above were considered high EdU.

Western blot

Cells growing exponentially at approximately 70% confluence were treated under the desired conditions. Cells were starved overnight, treated with the appropriate inhibitor and lysed with RIPA buffer enriched with phosphatase and protease inhibitors. Whole cell lysates were subjected to acrylamide SDS-PAGE, using standard procedures, then were transferred onto a nitrocellulose support membrane (Immobilon, Millipore) and western blotted. The primary antibodies at 1:1000 dilution were: P-ERK1/2 (Cell Signaling, Ref. 4370), ERK1/2 (Cell Signaling, Ref. 4695), P-AKT (Ser473, Cell Signaling, Ref. 9271), AKT (Cell Signaling, Ref. 9272), P-PRAS40 (Thr246, Cell Signaling, Ref. 2997), PRAS40 (Cell Signaling, Ref. 2691), P-S6 Ribosomal Protein (Ser235/236, Cell Signaling, Ref. 4858), S6 Ribosomal Protein (Cell Signaling Ref. 2217), P-MEK1/2 (Ser217/221, Cell Signaling, Ref. 9154), MEK1/2 (Cell Signaling, Ref. 8727) and αTubulin (Santa Cruz, Ref. 23948).. The secondary antibodies, all at 1:5,000 dilution, were: Goat-anti-mouse IgG, DyLight TM800 (Thermo Scientific, Ref: 35521) and goat-anti-rabbit IgG, DylightTM800 (Thermo Scientific, Ref: 10036). Finally, data were collected using an Odyssey Infrared imaging system (Li-Cor Biosciences) and quantified using Image J software.

PathScan intracellular signaling array assay

To detect the activation of intracellular signaling, SNU-449, Hep-G2, SNU-182, SNU-475, SNU-423 and HUH-7 cells were starved 3h and treated with the indicated IC_{50} concentrations of the inhibitors or the control vehicle (DMSO). After 1h of treatment, cells were collected and lysed. Intracellular signaling was detected using a PathScan® Intracellular Signaling Array Kit (Cell Signaling Technology, Ref. #7744) following the manufacturer's instructions. Data were collected using an Odyssey Infrared imaging system (Li-Cor Biosciences) and quantified using Image J software.

REFERENCES

1. Li H, Durbin R. Fast and accurate short read alignment with Burrows-Wheeler transform. Bioinformatics. 2009; 25:1754-60. https://doi.org/10.1093/bioinformatics/btp324.
2. McKenna A, Hanna M, Banks E, Sivachenko A, Cibulskis K, Kernytsky A, Garimella K, Altshuler D, Gabriel S, Daly M, DePristo MA. The genome analysis toolkit: a mapreduce framework for analyzing next-generation DNA sequencing data. Genome Res. 2010; 20:1297-303. https://doi.org/10.1101/gr.107524.110.
3. Martinez N, Almaraz C, Vaque JP, Varela I, Derdak S, Beltran S, Mollejo M, Campos-Martin Y, Agueda L, Rinaldi A, Kwee I, Gut M, Blanc J, et al. Whole-exome sequencing in splenic marginal zone lymphoma reveals mutations in genes involved in marginal zone differentiation. Leukemia. 2014; 28:1334-40. https://doi.org/10.1038/leu.2013.365.
4. Ye K, Schulz MH, Long Q, Apweiler R, Ning Z. Pindel: a pattern growth approach to detect break points of large deletions and medium sized insertions from paired-end short reads. Bioinformatics. 2009; 25:2865-71. https://doi.org/10.1093/bioinformatics/btp394.
5. Chou TC, Talalay P. Quantitative analysis of dose-effect relationships: the combined effects of multiple drugs or enzyme inhibitors. Adv Enzyme Regul. 1984; 22:27-55.

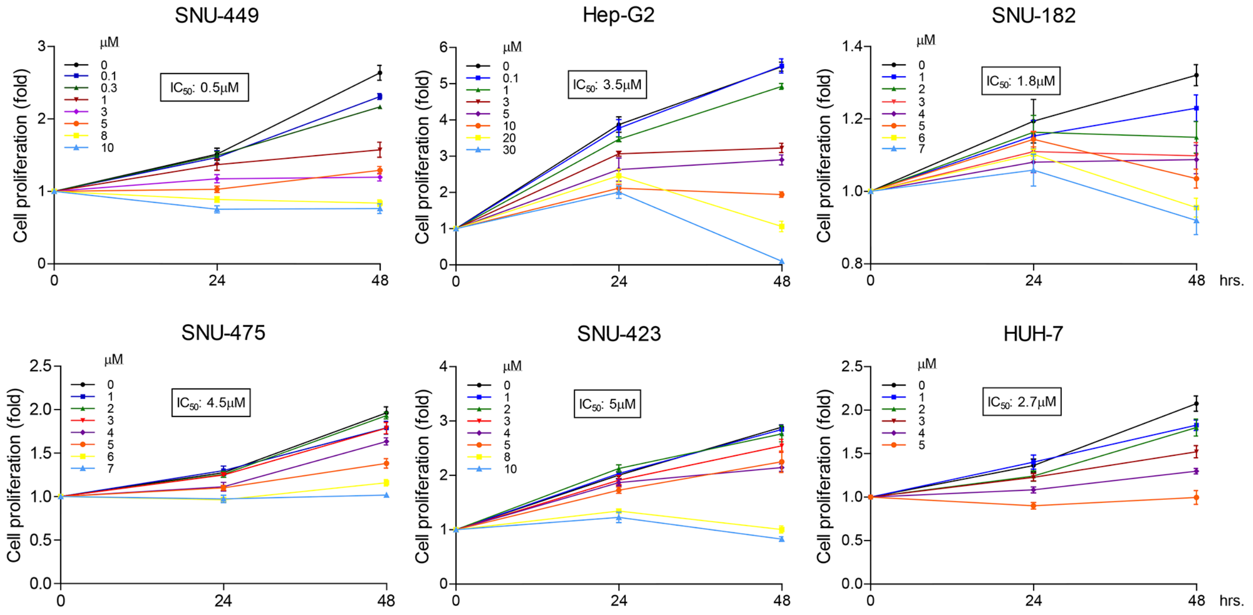

Supplementary Figure 1: IC_{50} values of sorafenib in HCC cell lines. Proliferation analysis of SNU-449, Hep-G2, SNU-182, SNU-475, SNU-423 and HUH-7 cells at 0, 24 and 48h treated with the indicated concentrations of sorafenib.

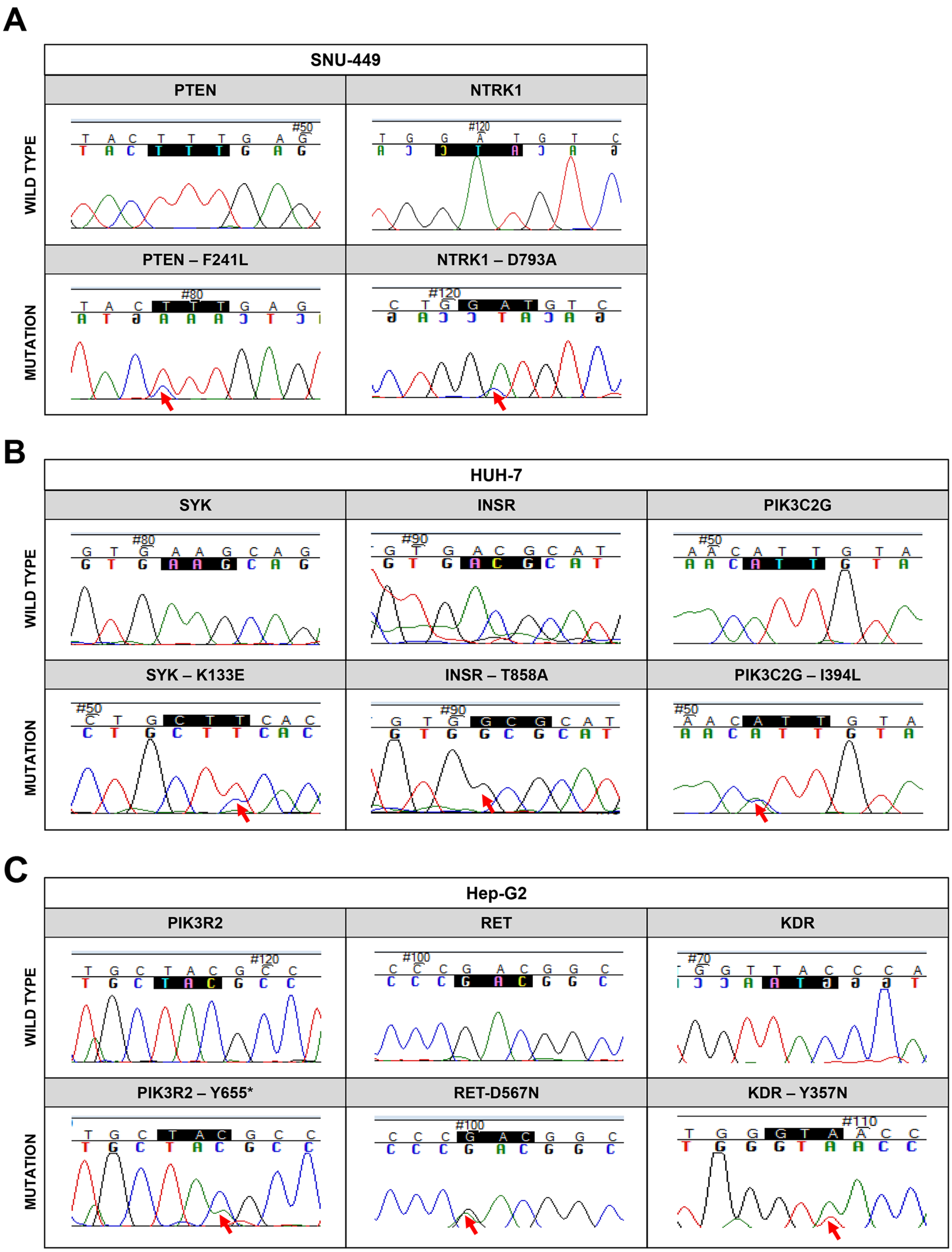

Supplementary Figure 2: Sanger validation of potentially actionable mutations found *in silico* in SNU-449, HUH-7 and Hep-G2 cells. Sanger sequencing of genomic DNA from **(A)** SNU-449, **(B)** HUH-7 and **(C)** Hep-G2 cells. Red arrow indicate the amino acid change.

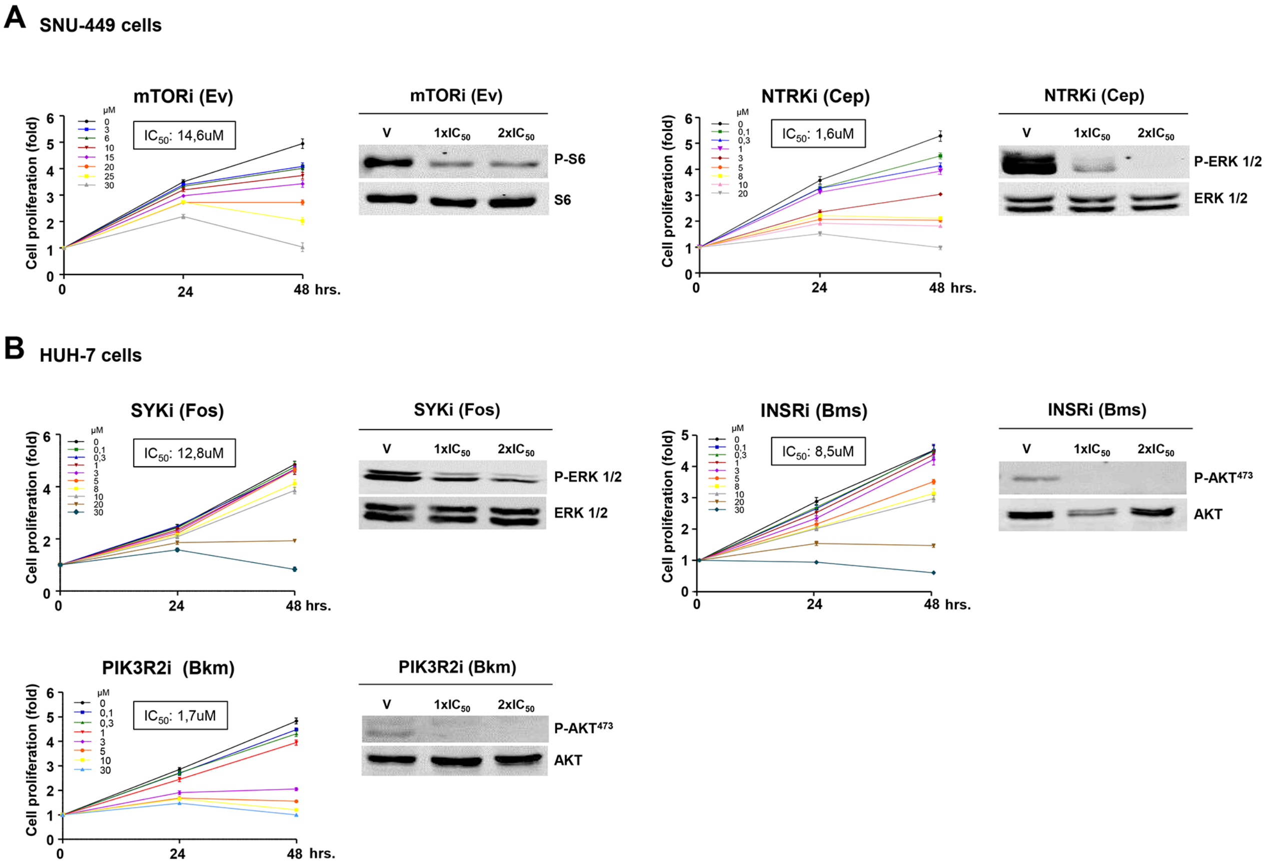

Supplementary Figure 3: IC_{50} values of mutation-guided inhibitors for HCC cell lines. Proliferation analysis of (A) SNU-449 and (B) HUH-7 cells at 0, 24 and 48h treated with the indicated concentrations of each inhibitor: mTORi (Ev: Everolimus) and NTRKi (Cep: Lestaurtinib) for SNU-449 cells and SYKi (Fos: Fostamatinib), INSRi (Bms: BMS-754807) and PIK3R2i (Bkm: BKM-120) for HUH-7 cells. The IC_{50} concentration of each inhibitor is calculated at 48h. Western Blotting analyses of **(A)** SNU-449 and **(B)** HUH-7 cells treated with control vehicle (V), 1x IC_{50} or 2x IC_{50} concentrations of each inhibitor and incubated with the indicated antibodies.

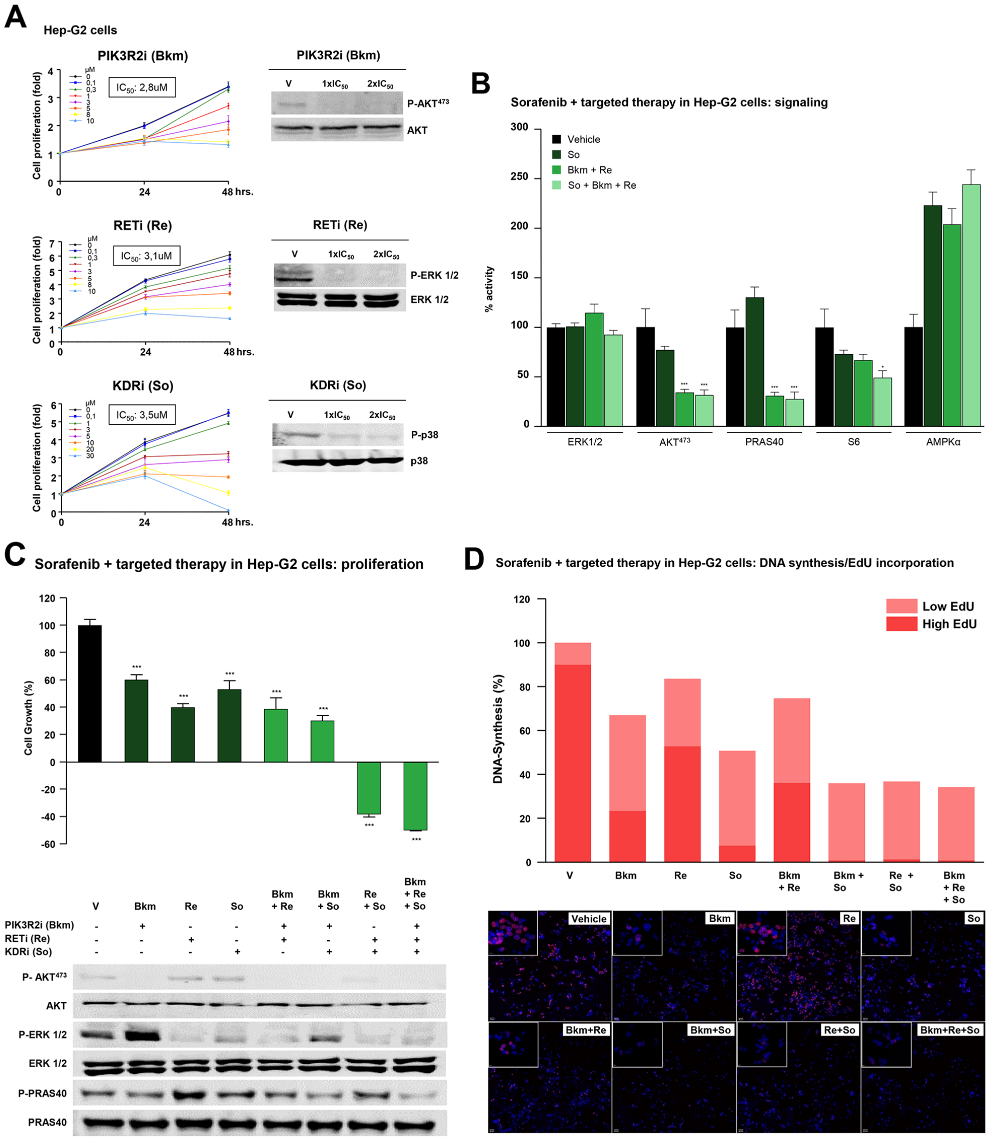

Supplementary Figure 4: Combination of sorafenib and targeted therapy in Hep-G2 cell line. **(A)** Proliferation analysis of Hep-G2 cells at 0, 24 and 48h treated with the indicated concentrations of each inhibitor: PIK3R2i (Bkm: Buparlisib), RETi (Re: Regorafenib) and KDRi (So: Sorafenib). Western Blotting analyses of Hep-G2 cells treated with control vehicle (V), 1x IC50 or 2x IC50 concentrations of each inhibitor and incubated with the indicated antibodies. **(B)** Intracellular signaling array of Hep-G2 cells starved and treated for 1h with control vehicle (black bar) or the IC_{50} concentrations of sorafenib (dark green bar) or combination of sorafenib with targeted inhibitors (light green bars), as indicated. **(C)** *Top:* Proliferation analysis of Hep-G2 cells at 48h incubated with control vehicle (V, black bar) or the IC_{50} concentration of the indicated inhibitor (PIK3R2i (Bkm: buparlisib), RETi (Re: regorafenib) and KDRi (So: sorafenib) alone (dark green bars), or in double or triple combination (light green bars). *Bottom:* Western Blotting analysis of Hep-G2 cells treated for 1h with control vehicle, or the indicated inhibitor, or the combination of inhibitors under the same conditions as above, and incubated with P-AKT^{473}, AKT, P-ERK1/2, ERK1/2, P-PRAS40 and PRAS40 antibodies, as indicated. **(D)** DNA synthesis assay using Click-iT® EdU in Hep-G2 cells incubated for 24h under the same conditions as in (C). Graph bars show percentage of low (light red) or high (intense red) EdU-stained cells in three photographic fields from a representative experiment. Representative pictures show the nucleus of the total number of cells (blue dots) and EdU-positive cells (red dots). Error bars show the SEM. $^{*}P \leq 0.05$; $^{**}P \leq 0.01$; $^{***}P \leq 0.001$.

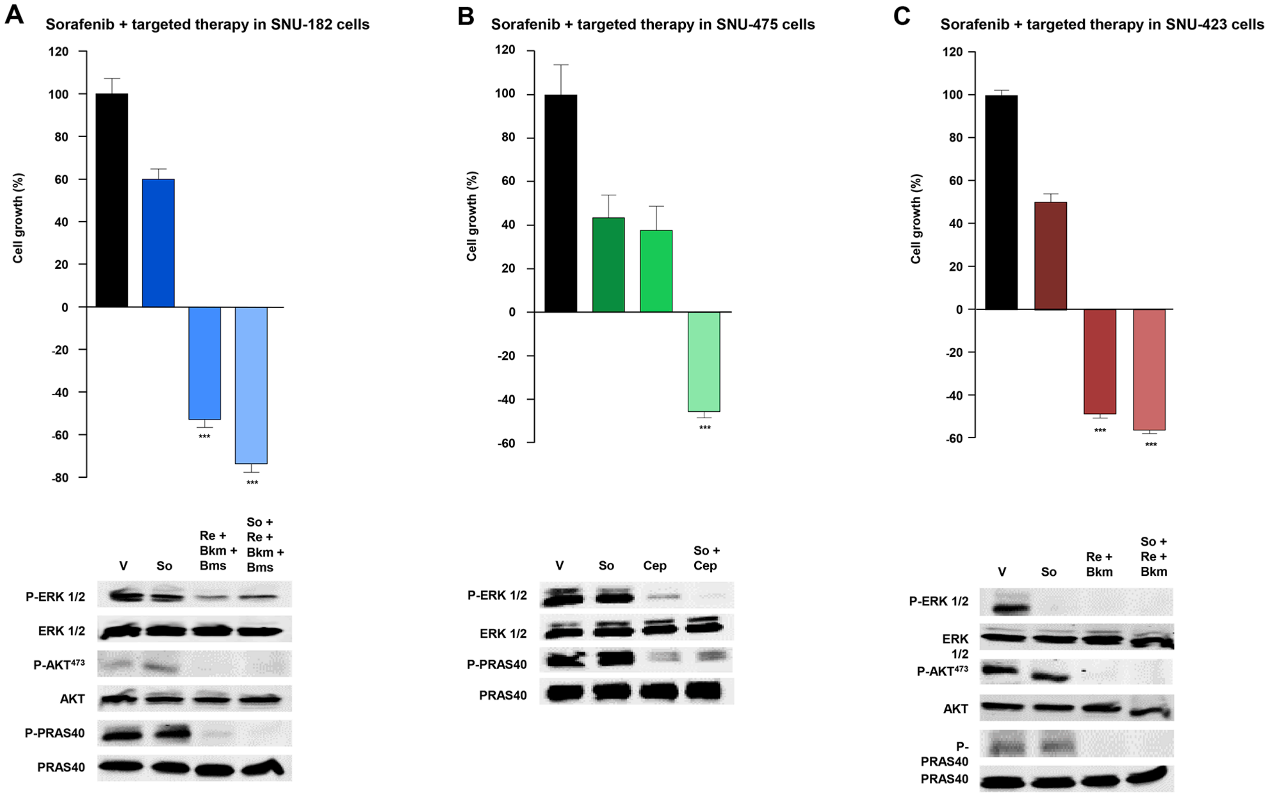

Supplementary Figure 5: Effects of sorafenib and targeted therapy in HCC cell lines. Proliferation analyses of SNU-182 (**A**, *top*), SNU-475 (**B**, *top*) and SNU-423 cells (**C**, *top*) after 48h of incubation with control vehicle (V) or the IC_{50} concentrations of sorafenib alone (second bar from the left), targeted inhibitors (third bar) or in combination with these (rightmost bar). Western blot analyses of SNU-182 (A, *bottom*), SNU-475 (B, *bottom*) and SNU-423 (C, *bottom*) cells starved, treated under the same conditions as above for 1h and incubated with P-ERK1/2, ERK1/2, P-AKT^{473}, AKT, P-PRAS40 and PRAS40 antibodies, as indicated. Statistical analyses show targeted therapy or sorafenib plus targeted therapy versus sorafenib alone. Error bars show the SEM. * $P \leq 0.05$; $^{**}P \leq 0.01$; $^{***}P \leq 0.001$.

A

		ED50	ED75	ED90
SNU-449	So + Cep	0,93	0,90	0,88
	So + Ev	0,80	0,53	0,44
HUH-7	So + Fos	0,81	0,43	0,23
	So + Bms	0,55	0,27	0,15
	So + Bkm	0,81	0,42	0,22

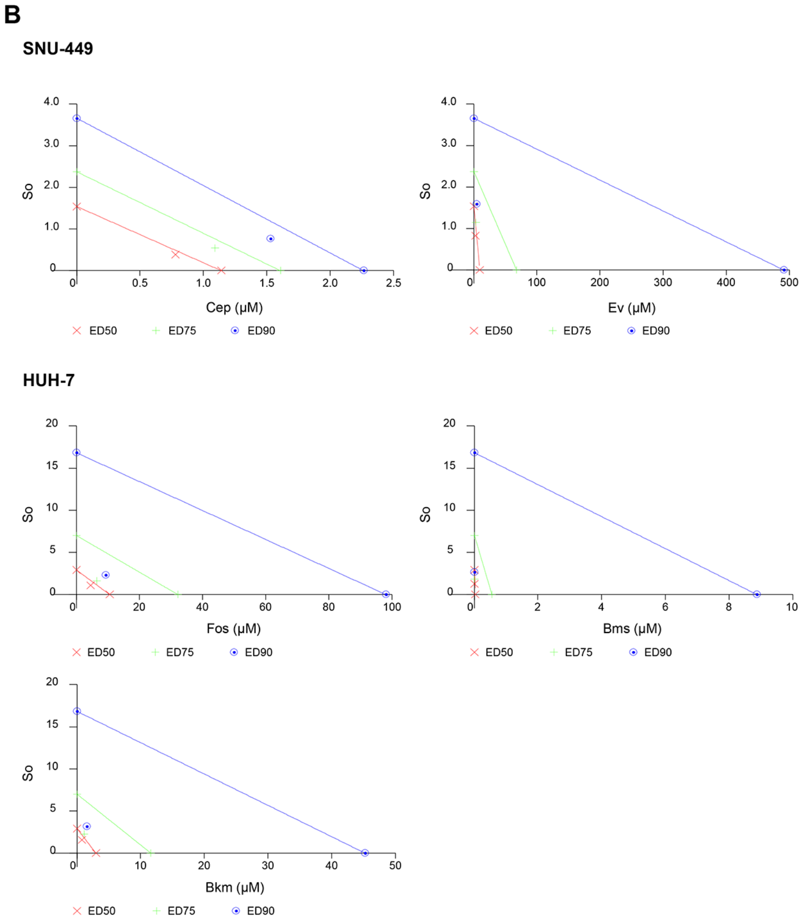

Supplementary Figure 6: Synergistic effects of sorafenib plus targeted inhibitors in HCC cells. (A) Combination indexes (CI) of sorafenib plus targeted therapies effects in SNU-449 and HUH-7 cells. CI<1 synergism; CI=1 additive effect and CI>1 antagonism. **(B)** Isobologram analyses for the interaction of various combinations of sorafenib (So) with everolimus (Ev) or lestaurtinib (Cep) in SNU-449 cells and sorafenib (So) with fostamatinib (Fos), BMS-754807 (Bms) or buparlisib (Bkm) in HUH-7 cells.

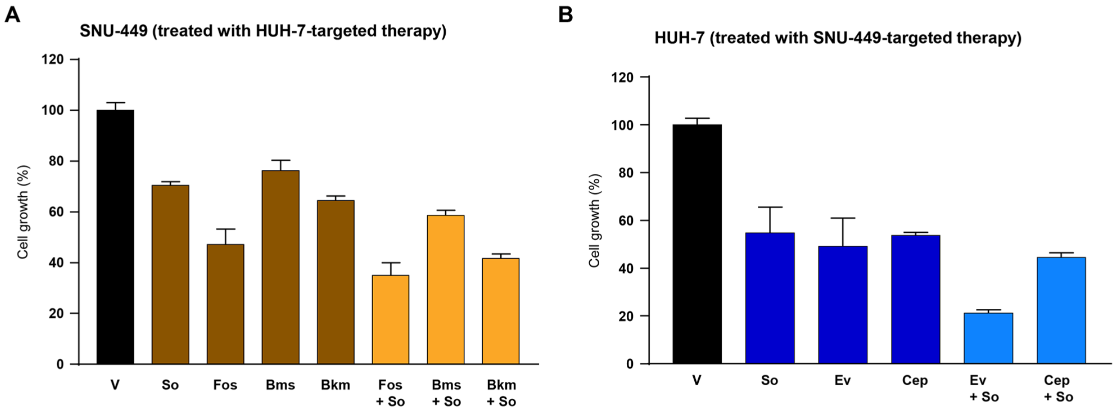

Supplementary Figure 7: Proliferation effects of sorafenib in combination with the interchanged specific inhibitors in SNU-449 and HUH-7 cells. Proliferation analyses of SNU-449 **(A)** and HUH-7 cells **(B)** at 48h incubated with control vehicle (V) or the IC_{50} concentrations of each inhibitor alone (dark brown and dark blue bars, respectively) or in double combination with sorafenib (light brown and light blue bars, respectively).

Supplementary Table 1: HCC cases with somatic mutations studied *in silico.* Meta-analysis showing the number and percentage of cases found to be mutated with the potential to guide targeted therapy

Cohort	Reference	Analysis	Cases	Nº cases with mutations (%)
**Design**	Huang, J., et al., Nat Genet, 2012	WES	10	9 (90)
	Guichard, C., et al., Nat Genet, 2012	WES	24	24 (100)
	Broad Institute, Cancer Cell Line Encyclopaedia	WGS	7	7 (100)
**Validation**	Schulze, K., et al., Nat Genet, 2015	WES	243	172 (70,8)
	Kan, Z., et al., Genome Res, 2013	WGS	88	57 (64,7)

Supplementary Table 2: Number of hits detected *in silico*. Total genes and number of hits detected *in silico* in the validation cohort. The genes are grouped in signaling pathways or functional families.

See Supplementary File 1

Supplementary Table 3: Clinical characteristics of the patients studied in the discovery cohort. The table shows the clinical characterization of the lesions from 32 initial hepatocellular carcinoma patients included retrospectively and prospectively in our mutational study: gender, age at diagnosis, etiology, radiology state, BCLC index (Barcelona Clinic Liver Cancer; 0 very early stage; A early stage and B intermediate stage) and origin of sample (R: resection and T: transplant)

Patient	Gender	Age	Etiology	Radiology state	BCLC	Sample type
**P01**	M	60	Alcohol	One nodule 1,5cm	0	R
**P02**	M	64	Alcohol	One nodule 4cm	A	R
**P03**	M	70	Alcohol	One nodule 2cm	A	R
**P04**	F	56	Alcohol	One nodule 2,2cm	A	R
**P05**	F	68	Alcohol	One nodule 2cm	0	R
**P06**	M	73	Alcohol	One nodule 4,3cm	A	R
**P07**	M	60	Alcohol	Two nodules 2,4 and 1cm	A	R
**P08**	M	63	Alcohol	One nodule 3cm	A	R
**P09**	M	58	Alcohol	One nodule 4,7cm	A	R
**P10**	M	69	Alcohol	One nodule 8cm	B	R
**P11**	M	64	Alcohol	One nodule 1,9cm	A	T
**P12**	M	66	Alcohol	One nodule 2,7cm	A	R
**P13**	M	46	HBV	One nodule more than 5cm	B	R
**P14**	M	65	HBV	Two nodules 1,5 and 1,7cm	A	T
**P15**	M	80	HBV	One nodule 8cm	B	R
**P16**	M	60	HBV+Alcohol	One nodule 5,1cm	B	R
**P17**	M	59	HCV	One nodule 3cm	A	T
**P18**	M	67	HCV	One nodule 2cm	0	R
**P19**	M	53	HCV	One nodule 3cm	A	T
**P20**	M	57	HCV	One nodule more than 5cm	B	R
**P21**	M	62	HCV	One nodule 3,3cm	A	R
**P22**	M	59	HCV	One nodule 4,5cm	A	R
**P23**	M	76	HCV	One nodule 1,6cm	A	R
**P24**	M	60	HCV	One nodule 3,5cm	A	R
**P25**	M	77	HCV	One nodule 4,5cm	A	R
**P26**	M	56	HCV	One nodule 2,5cm	A	R
**P27**	M	55	HCV	One nodule 1cm	A	R
**P28**	M	76	HCV+Alcohol	One nodule 2,5cm	A	R
**P29**	F	57	HCV+Alcohol	One nodule 4cm	A	R
**P30**	M	66	Hemochromatosis	One nodule 2,7cm	A	R
**P31**	M	59	Hemochromatosis	One nodule 2,4cm	A	R
**P32**	M	73	Hemochromatosis	One nodule 4cm	A	R

Supplementary Table 4: Somatic mutations detected *in silico* in HCC cells lines with a known mutational profile. Table showing the mutational characteristics of six commercial cell lines (*in silico* comparison with CCLE data) and the IC_{50} concentration of sorafenib. Cell line: Cell line name; Chr.: Chromosome number; Position: Genomic location of the mutation in the chromosome; AA change: Amino acid change; Gene: Gene name and IC_{50} sorafenib (µM): Micromolar IC_{50} concentration of sorafenib.

See Supplementary File 2

Supplementary Table 5: Mean and SEM of the 18 antibodies included in the PathScan® Intracellular Signaling Array Kit for the Hep-G2, SNU-449, HUH-7, SNU-182, SNU-475 and SNU-423 cell lines.

See Supplementary File 3

Supplementary Table 6: Oligonucleotides used to validate panel and cell lines. The table shows the genes and genomic locations studied to validate mutations found in our validation cohort and in cell lines.

See Supplementary File 4

Supplementary Table 7: Effects of case-specific targeted therapies over proliferation and intracellular signaling in HCC cells. Table shows mean percentages of proliferation and phosphorylation statuses (data obtained from PathScan® Intracellular Signaling Array Kit) with respect to controls (100%) in each cell line

Cell line	Proliferation	Activity					
		P-ERK1/2	P-AKT^{473}	P-GSK-3β	P-S6	P-PRAS40	P-AMPKα
**SNU-449**	38,58	114,71	34,10	74,59	66,69	30,58	203,83
**Hep-G2**	37,60	77,02	43,63	22,89	14,89	20,63	111,20
**SNU-182**	12,46	70,87	29,77	55,82	14,03	4,48	97,93
**SNU-475**	-40,83	69,07	36,77	62,52	18,22	13,28	100,81
**SNU-423**	-48,21	57,39	97,17	40,77	7,85	18,01	152,37
**HUH-7**	-52,91	95,08	92,46	40,20	9,47	30,82	193,53
**Mean (overall)**	-8,88	80,69	55,65	49,47	21,86	19,63	143,28
**SD**	43,30	20,75	30,71	18,49	22,28	10,18	47,25
**EE**	17,68	8,47	12,54	7,55	9,10	4,16	19,29
**95%CI Upper limit**	36,56	102,46	87,89	68,87	45,24	30,32	192,87
**95%CI Lower limit**	-54,32	58,92	23,43	30,06	-1,52	8,95	93,69
**p value (H0 ≠ 100)**	0,0016	0,0715	0,0166	0,0011	0,0004	<0,0001	0,0749
