# Supplementary material for: Applied diagnostics in liver cancer. Efficient combinations of sorafenib with targeted inhibitors blocking AKT/mTOR
Source: Oncotarget. 2018 Jul 20;9(56):30869–82. doi: 10.18632/oncotarget.25766 (PMC6089396; doi:10.18632/oncotarget.25766)
Supplement: Supplementary file 2 [file oncotarget-09-30869-s002.docx]

**Supplementary Table 2:** Number of hits detected *in silico*. Total genes and number of hits detected *in silico* in the validation cohort. The genes are grouped in signaling pathways or functional families.

| **Gene** | **Hits** | **Signaling pathway** |
| --- | --- | --- |
| ABCA5 | 3 | OTHER |
| ALDH2 | 3 | METHABOLISM |
| APC | 8 | WNT |
| ATM | 13 | CHROMATIN REGULATOR AND REPAIR |
| ATP6V1A | 1 | OTHER |
| ATR | 8 | CHROMATIN REGULATOR AND REPAIR |
| AURKA | 2 | OTHER |
| AXIN1 | 27 | WNT |
| BRAF | 2 | MAPK |
| BRDT | 3 | CHROMATIN REGULATOR AND REPAIR |
| CAMKK2 | 4 | CA+2 |
| CASP4 | 2 | OTHER |
| CCKAR | 2 | GPCR |
| CPT1A | 2 | METHABOLISM |
| CSF1R | 2 | RTK |
| CSNK1G3 | 1 | WNT |
| CTNNB1 | 117 | WNT |
| DDR1 | 1 | GPCR |
| DRD3 | 3 | GPCR |
| EDN3 | 2 | GPCR |
| EGF | 2 | RTK |
| EGFR | 5 | RTK |
| EIF4E | 1 | OTHER |
| EIF4EBP1 | 1 | OTHER |
| ELK3 | 1 | CHROMATIN REGULATOR AND REPAIR |
| FGFR1 | 1 | RTK |
| FGFR2 | 3 | RTK |
| FLT1 | 8 | RTK |
| FLT3 | 5 | RTK |
| GLI1 | 5 | CHROMATIN REGULATOR AND REPAIR |
| HIF1A | 4 | PI3K/MTOR |
| HNF1A | 16 | CHROMATIN REGULATOR AND REPAIR |
| HRAS | 1 | MAPK |
| HSP90AB1 | 1 | OTHER |
| IDE | 4 | METHABOLISM |
| INSR | 5 | RTK |
| ITPR1 | 5 | CA+2 |
| ITPR2 | 7 | Ca+2 |
| JAK1 | 8 | JAK STAT |
| JAK3 | 5 | JAK STAT |
| KDR | 7 | RTK |
| MAP2K1 | 2 | MAPK |
| MAP4K3 | 1 | MAPK |
| MAPK8 | 2 | MAPK |
| MAPKAP1 | 1 | PI3K/MTOR |
| MET | 3 | RTK |
| MLST8 | 1 | PI3K/MTOR |
| MMACHC | 2 | METHABOLISM |
| MMP1 | 3 | OTHER |
| MPO | 3 | METHABOLISM |
| MTOR | 5 | PI3K/MTOR |
| MYH1 | 7 | CYTOSKELETON |
| MYH10 | 3 | CYTOSKELETON |
| MYH9 | 4 | CYTOSKELETON |
| MYLK | 6 | CYTOSKELETON |
| NOTCH1 | 6 | OTHER |
| NOV | 3 | OTHER |
| NTRK1 | 2 | RTK |
| PAM | 4 | METHABOLISM |
| PGR | 7 | OTHER |
| PIK3C2G | 4 | PI3K/MTOR |
| PIK3CA | 5 | PI3K/MTOR |
| PIK3CB | 1 | PI3K/MTOR |
| PIK3CG | 5 | PI3K/MTOR |
| PIK3R2 | 3 | PI3K/MTOR |
| PIK3R4 | 6 | PI3K/MTOR |
| PLA2G6 | 5 | OTHER |
| PPARG | 1 | OTHER |
| PPP2R1B | 1 | OTHER |
| PRKAA2 | 1 | METHABOLISM |
| PRKAR1B | 1 | METHABOLISM |
| PRKCQ | 1 | OTHER |
| PRKDC | 7 | CHROMATIN REGULATOR AND REPAIR |
| PRR5 | 1 | PI3K/MTOR |
| PSENEN | 1 | OTHER |
| PTEN | 6 | PI3K/MTOR |
| PTPRS | 7 | OTHER |
| RAC1 | 1 | MAPK |
| RAF1 | 3 | MAPK |
| RET | 4 | RTK |
| RICTOR | 2 | PI3K/MTOR |
| SCN3A | 8 | OTHER |
| SLC10A1 | 3 | OTHER |
| SLC16A1 | 2 | OTHER |
| SLCO4A1 | 2 | METHABOLISM |
| SYK | 5 | OTHER |
| TGFB1 | 1 | OTHER |
| TP53 | 95 | P53 |
| TRPV5 | 3 | CA+2 |
| TSC1 | 6 | PI3K/MTOR |
| TSC2 | 7 | PI3K/MTOR |
| TYRO3 | 4 | RTK |
| VCAM1 | 3 | OTHER |
