# Supplementary material for: Applied diagnostics in liver cancer. Efficient combinations of sorafenib with targeted inhibitors blocking AKT/mTOR
Source: Oncotarget. 2018 Jul 20;9(56):30869–82. doi: 10.18632/oncotarget.25766 (PMC6089396; doi:10.18632/oncotarget.25766)
Supplement: Supplementary file 3 [file oncotarget-09-30869-s003.docx]

**Supplementary Table 4:** Somatic mutations detected *in silico* in HCC cells lines with a known mutational profile. Table showing the mutational characteristics of six commercial cell lines (*in silico* comparison with CCLE data) and the IC_50_ concentration of sorafenib. Cell line: Cell line name; Chr.: Chromosome number; Position: Genomic location of the mutation in the chromosome; AA change: Amino acid change; Gene: Gene name and IC_50_ sorafenib (µM): Micromolar IC_50_ concentration of sorafenib.

| **Cell line** | **Chr.** | **Position** | **AA change** | **Gene** | **IC_50_ Sorafenib (μM)** |
| --- | --- | --- | --- | --- | --- |
| **Hep-G2** | 1 | 92446270 | E457A | **BRDT** | 3,5 |
|  | 2 | 39485723 | P772S | **MAP4K3** |  |
|  | 4 | 55976843 | Y357N | **KDR** |  |
|  | 10 | 43608351 | D567N | **RET** |  |
|  | 19 | 18279692 | Y655* | **PIK3R2** |  |
|  | 22 | 36702015 | G707D | **MYH9** |  |
| **SNU-449** | 1 | 156851421 | D793A | **NTRK1** | 0,5 |
|  | 3 | 123337614 | E1790del | **MYLK** |  |
|  | 3 | 123457877 | R152H | **MYLK** |  |
|  | 8 | 48771415 | G2113del | **PRKDC** |  |
|  | 10 | 89717696 | F241L | **PTEN** |  |
|  | 11 | 102667510 | D170E | **MMP1** |  |
|  | 11 | 104821821 | Y126C | **CASP4** |  |
|  | 12 | 121434456 | S407L | **HNF1A** |  |
|  | 16 | 343540 | R712* | **AXIN1** |  |
|  | 17 | 7578449 | A161T | **TP53** |  |
| **HUH-7** | 9 | 93606577 | K133E | **SYK** | 2,7 |
|  | 9 | 139417493 | Q184R | **NOTCH1** |  |
|  | 12 | 18762561 | I1394L | **PIK3C2G** |  |
|  | 17 | 7578190 | Y220C | **TP53** |  |
|  | 17 | 8396332 | T1407I | **MYH10** |  |
|  | 17 | 10411801 | N592K | **MYH1** |  |
|  | 19 | 7141798 | T858A | **INSR** |  |
|  | 22 | 36697093 | E881V | **MYH9** |  |
| **SNU-475** | 11 | 100912818 | L835Q | **PGR** | 4,5 |
|  | 13 | 28611336 | T432M | **FLT3** |  |
|  | 17 | 7577153 | G262D | **TP53** |  |
|  | 17 | 7577566 | N239D | **TP53** |  |
| **SNU-423** | 3 | 130452809 | V345F | **PIK3R4** | 5 |
|  | 6 | 44219997 | V575A | **HSP90AB1** |  |
|  | 12 | 57865443 | F974V | **GLI1** |  |
|  | 13 | 28913428 | E789K | **FLT1** |  |
|  | 17 | 7578556 | Y126_splice | **TP53** |  |
|  | 20 | 54956539 | Y219fs | **AURKA** |  |
|  | 20 | 61288032 | R76W | **SLCO4A1** |  |
| **SNU-182** | 3 | 123456362 | R206H | **MYLK** | 1,8 |
|  | 3 | 130409498 | R1033S | **PIK3R4** |  |
|  | 13 | 29008268 | E201D | **FLT1** |  |
|  | 17 | 7578205 | S215I | **TP53** |  |
|  | 17 | 10417137 | F247_splice | **MYH1** |  |
|  | 19 | 7184495 | P269L | **INSR** |  |
