# Supplementary material for: Applied diagnostics in liver cancer. Efficient combinations of sorafenib with targeted inhibitors blocking AKT/mTOR
Source: Oncotarget. 2018 Jul 20;9(56):30869–82. doi: 10.18632/oncotarget.25766 (PMC6089396; doi:10.18632/oncotarget.25766)
Supplement: Supplementary file 4 [file oncotarget-09-30869-s004.docx]

**Supplementary Table 5:** Mean and SEM of the 18 antibodies included in the PathScan® Intracellular Signaling Array Kit for the Hep-G2, SNU-449, HUH-7, SNU-182, SNU-475 and SNU-423 cell lines.

|  | **Hep-G2** | | | | | | | | |
| --- | --- | --- | --- | --- | --- | --- | --- | --- | --- |
|  | **Vehicle** | | **So** | | **Bkm + Re** | | **So + Bkm + Re** | | |
| **Target** | **Mean** | **SEM** | **Mean** | **SEM** | **Mean** | **SEM** | **Mean** | **SEM** | |
| **Erk1/2** | **100,00** | 3,85 | **100,87** | 3,67 | **114,71** | 8,95 | **92,44** | 4,72 |  |
| **Stat1** | **100,00** | 6,40 | **90,62** | 8,31 | **95,55** | 7,76 | **95,11** | 9,75 |  |
| **Stat3** | **100,00** | 4,20 | **119,54** | 9,34 | **120,81** | 11,30 | **119,39** | 12,10 |  |
| **Akt**^308^ | **100,00** | 14,28 | **120,55** | 4,19 | **93,33** | 2,21 | **85,51** | 5,27 |  |
| **Akt**^473^ | **100,00** | 18,92 | **77,08** | 4,02 | **34,10** | 3,61 | **31,59** | 5,25 |  |
| **AMPK**α | **100,00** | 13,47 | **223,00** | 13,62 | **203,83** | 16,04 | **244,32** | 14,84 |  |
| **S6 Ribosomal Protein** | **100,00** | 18,63 | **72,78** | 4,36 | **66,69** | 6,16 | **49,06** | 7,30 |  |
| **mTOR** | **100,00** | 12,06 | **104,58** | 4,76 | **97,02** | 8,25 | **87,94** | 5,63 |  |
| **HSP27** | **100,00** | 8,08 | **114,46** | 3,67 | **105,54** | 2,72 | **109,58** | 4,67 |  |
| **Bad** | **100,00** | 6,17 | **144,35** | 2,47 | **152,09** | 9,06 | **135,29** | 3,18 |  |
| **p70 S6 Kinase** | **100,00** | 12,66 | **96,93** | 6,22 | **110,82** | 9,50 | **88,78** | 5,32 |  |
| **PRAS40** | **100,00** | 17,74 | **130,09** | 10,69 | **30,58** | 4,03 | **27,32** | 7,59 |  |
| **p53** | **100,00** | 6,95 | **114,30** | 5,37 | **125,12** | 14,26 | **123,56** | 7,38 |  |
| **p38** | **100,00** | 8,14 | **97,48** | 2,11 | **117,84** | 10,66 | **106,18** | 4,91 |  |
| **SAPK/JNK** | **100,00** | 6,60 | **92,93** | 4,98 | **93,17** | 6,55 | **91,10** | 4,67 |  |
| **PARP** | **100,00** | 4,24 | **104,57** | 4,48 | **101,16** | 5,55 | **101,81** | 8,12 |  |
| **Caspase-3** | **100,00** | 6,17 | **101,11** | 1,58 | **105,09** | 5,77 | **107,38** | 6,75 |  |
| **GSK-3**β | **100,00** | 4,60 | **84,87** | 4,68 | **74,59** | 5,13 | **72,30** | 4,90 |  |

|  | **SNU-449** | | | | | | | |
| --- | --- | --- | --- | --- | --- | --- | --- | --- |
|  | **Vehicle** | | **So** | | **Ev + Cep** | | **So + Ev + Cep** | |
| **Target** | **Mean** | **SEM** | **Mean** | **SEM** | **Mean** | **SEM** | **Mean** | **SEM** |
| **Erk1/2** | **100,00** | 3,87 | **105,71** | 5,27 | **70,87** | 4,77 | **86,08** | 6,00 |
| **Stat1** | **100,00** | 5,04 | **127,83** | 10,57 | **100,44** | 7,81 | **109,39** | 7,44 |
| **Stat3** | **100,00** | 5,52 | **117,78** | 3,59 | **100,52** | 3,06 | **105,36** | 5,67 |
| **Akt**^308^ | **100,00** | 4,03 | **113,22** | 6,09 | **64,71** | 2,94 | **81,38** | 10,27 |
| **Akt**^473^ | **100,00** | 6,19 | **106,12** | 9,04 | **29,77** | 1,92 | **50,75** | 8,38 |
| **AMPK**α | **100,00** | 1,92 | **146,53** | 8,20 | **97,93** | 12,08 | **129,59** | 26,25 |
| **S6 Ribosomal Protein** | **100,00** | 3,73 | **47,55** | 5,99 | **14,03** | 0,90 | **23,81** | 5,46 |
| **mTOR** | **100,00** | 2,40 | **114,98** | 6,13 | **88,20** | 6,91 | **98,01** | 4,08 |
| **HSP27** | **100,00** | 3,36 | **119,97** | 8,09 | **90,57** | 4,71 | **95,35** | 8,41 |
| **Bad** | **100,00** | 1,86 | **120,83** | 5,76 | **88,86** | 9,56 | **107,38** | 4,65 |
| **p70 S6 Kinase** | **100,00** | 9,58 | **119,10** | 7,24 | **79,94** | 5,19 | **110,21** | 8,46 |
| **PRAS40** | **100,00** | 7,13 | **98,00** | 9,25 | **4,48** | 0,36 | **16,28** | 7,06 |
| **p53** | **100,00** | 5,32 | **108,50** | 5,19 | **86,14** | 6,01 | **96,58** | 5,79 |
| **p38** | **100,00** | 1,46 | **99,92** | 3,56 | **98,65** | 7,34 | **96,76** | 6,02 |
| **SAPK/JNK** | **100,00** | 3,97 | **114,72** | 10,76 | **69,84** | 3,67 | **88,55** | 1,26 |
| **PARP** | **100,00** | 12,27 | **121,07** | 5,92 | **146,07** | 11,82 | **138,85** | 4,51 |
| **Caspase-3** | **100,00** | 5,34 | **114,71** | 3,76 | **112,14** | 5,33 | **119,26** | 8,63 |
| **GSK-3**β | **100,00** | 4,50 | **107,26** | 6,83 | **55,82** | 3,04 | **66,18** | 5,67 |

|  | **HUH-7** | | | | | | | |
| --- | --- | --- | --- | --- | --- | --- | --- | --- |
|  | **Vehicle** | | **So** | | **Fos + Bms + Bkm** | | **So + Fos + Bms + Bkm** | |
| **Target** | **Mean** | **SEM** | **Mean** | **SEM** | **Mean** | **SEM** | **Mean** | **SEM** |
| **Erk1/2** | **100,00** | 5,07 | **64,26** | 6,28 | **69,07** | 4,52 | **65,65** | 10,65 |
| **Stat1** | **100,00** | 8,23 | **104,33** | 17,17 | **90,81** | 6,39 | **90,85** | 9,35 |
| **Stat3** | **100,00** | 4,25 | **101,41** | 5,99 | **100,29** | 4,42 | **107,78** | 9,09 |
| **Akt**^308^ | **100,00** | 9,01 | **103,87** | 11,83 | **76,72** | 4,97 | **79,65** | 3,78 |
| **Akt**^473^ | **100,00** | 5,38 | **102,57** | 16,98 | **36,77** | 4,71 | **37,46** | 2,41 |
| **AMPK**α | **100,00** | 2,61 | **133,28** | 25,25 | **100,81** | 3,05 | **196,42** | 11,87 |
| **S6 Ribosomal Protein** | **100,00** | 4,03 | **27,28** | 6,46 | **18,22** | 1,12 | **16,63** | 0,70 |
| **mTOR** | **100,00** | 6,48 | **105,40** | 11,10 | **94,59** | 5,73 | **97,21** | 6,27 |
| **HSP27** | **100,00** | 12,99 | **103,65** | 19,62 | **102,07** | 10,47 | **105,26** | 7,58 |
| **Bad** | **100,00** | 6,72 | **95,08** | 14,08 | **96,39** | 7,60 | **99,03** | 7,78 |
| **p70 S6 Kinase** | **100,00** | 3,03 | **69,72** | 5,80 | **79,66** | 4,43 | **68,13** | 4,31 |
| **PRAS40** | **100,00** | 5,53 | **68,46** | 12,58 | **13,28** | 2,05 | **16,40** | 1,59 |
| **p53** | **100,00** | 14,72 | **94,41** | 16,90 | **103,22** | 17,11 | **99,93** | 9,33 |
| **p38** | **100,00** | 7,24 | **87,96** | 11,42 | **95,02** | 7,17 | **89,46** | 7,59 |
| **SAPK/JNK** | **100,00** | 4,63 | **86,68** | 6,14 | **92,84** | 7,60 | **82,04** | 3,93 |
| **PARP** | **100,00** | 13,25 | **106,98** | 15,52 | **99,83** | 15,96 | **96,84** | 17,82 |
| **Caspase-3** | **100,00** | 17,90 | **99,35** | 18,95 | **99,69** | 15,32 | **96,96** | 16,19 |
| **GSK-3**β | **100,00** | 11,07 | **82,04** | 12,95 | **62,52** | 8,03 | **62,49** | 6,04 |

|  | **SNU-182** | | | | **SNU-475** | | | | **SNU-423** | | | |
| --- | --- | --- | --- | --- | --- | --- | --- | --- | --- | --- | --- | --- |
|  | **Vehicle** | | **Re + Bkm + Bms** | | **Vehicle** | | **Cep** | | **Vehicle** | | **Re + Bkm** | |
| **Target** | **Mean** | **SEM** | **Mean** | **SEM** | **Mean** | **SEM** | **Mean** | **SEM** | **Mean** | **SEM** | **Mean** | **SEM** |
| **Erk1/2** | **100,00** | 3,95 | **95,08** | 5,51 | **100,00** | 6,97 | **77,02** | 6,34 | **100,00** | 5,16 | **57,39** | 1,35 |
| **Stat1** | **100,00** | 8,96 | **105,75** | 10,80 | **100,00** | 9,28 | **114,59** | 11,76 | **100,00** | 9,14 | **102,80** | 18,53 |
| **Stat3** | **100,00** | 6,96 | **121,15** | 8,04 | **100,00** | 9,88 | **104,61** | 12,10 | **100,00** | 13,56 | **93,43** | 5,17 |
| **Akt**^308^ | **100,00** | 4,06 | **97,79** | 4,81 | **100,00** | 2,28 | **82,67** | 7,70 | **100,00** | 11,57 | **96,49** | 5,17 |
| **Akt**^473^ | **100,00** | 6,27 | **92,46** | 9,53 | **100,00** | 5,10 | **43,63** | 3,25 | **100,00** | 12,03 | **97,17** | 16,45 |
| **AMPK**α | **100,00** | 2,31 | **193,53** | 3,24 | **100,00** | 4,50 | **111,20** | 6,43 | **100,00** | 4,48 | **152,37** | 3,87 |
| **S6 Ribosomal Protein** | **100,00** | 2,51 | **9,47** | 4,06 | **100,00** | 1,71 | **14,89** | 3,89 | **100,00** | 6,97 | **7,85** | 0,88 |
| **mTOR** | **100,00** | 5,88 | **89,87** | 4,19 | **100,00** | 6,66 | **104,88** | 6,35 | **100,00** | 6,41 | **77,24** | 3,51 |
| **HSP27** | **100,00** | 5,27 | **54,09** | 0,69 | **100,00** | 9,46 | **90,92** | 7,00 | **100,00** | 2,83 | **82,07** | 0,60 |
| **Bad** | **100,00** | 4,03 | **101,72** | 2,68 | **100,00** | 1,99 | **87,98** | 5,85 | **100,00** | 6,50 | **85,97** | 1,68 |
| **p70 S6 Kinase** | **100,00** | 3,73 | **83,08** | 5,19 | **100,00** | 2,30 | **96,48** | 1,28 | **100,00** | 4,07 | **102,03** | 4,17 |
| **PRAS40** | **100,00** | 2,83 | **30,82** | 0,76 | **100,00** | 3,84 | **20,63** | 1,29 | **100,00** | 2,72 | **18,01** | 0,57 |
| **p53** | **100,00** | 100,00 | **67,33** | 67,33 | **100,00** | 3,33 | **168,59** | 8,13 | **N/A** | N/A | **N/A** | N/A |
| **p38** | **100,00** | 25,49 | **120,38** | 18,94 | **100,00** | 8,33 | **69,34** | 14,41 | **100,00** | 19,47 | **38,02** | 14,79 |
| **SAPK/JNK** | **100,00** | 6,90 | **103,23** | 7,43 | **100,00** | 7,32 | **98,66** | 10,09 | **100,00** | 8,21 | **90,26** | 7,21 |
| **PARP** | **100,00** | 6,58 | **107,24** | 5,64 | **100,00** | 8,01 | **109,84** | 8,65 | **100,00** | 2,31 | **92,38** | 1,38 |
| **Caspase-3** | **100,00** | 4,00 | **108,62** | 4,00 | **100,00** | 6,91 | **109,87** | 8,41 | **100,00** | 8,22 | **86,29** | 3,65 |
| **GSK-3**β | **100,00** | 3,85 | **40,20** | 1,33 | **100,00** | 5,04 | **22,89** | 1,26 | **100,00** | 4,67 | **40,77** | 1,51 |
