# Supplementary material for: Applied diagnostics in liver cancer. Efficient combinations of sorafenib with targeted inhibitors blocking AKT/mTOR
Source: Oncotarget. 2018 Jul 20;9(56):30869–82. doi: 10.18632/oncotarget.25766 (PMC6089396; doi:10.18632/oncotarget.25766)
Supplement: Supplementary file 5 [file oncotarget-09-30869-s005.docx]

**Supplementary Table 6:** Oligonucleotides used to validate panel and cell lines. The table shows the genes and genomic locations studied to validate mutations found in our validation cohort and in cell lines.

| **Patient/cell line** | **Chr.** | **Position** | **Left primer** | **Right primer** |
| --- | --- | --- | --- | --- |
| **P-01** | 2 | 39605221 | AGTTCCCCTTCCTCCCCCAA | GGCACGGAATGTTAACACTGGT |
|  | 3 | 12627258 | TGCTGAGAACCACTCCAGCG | AGTGCAGAGAGGCTGTGACA |
|  | 17 | 7574003 | ATGAGATGGGGTCAGCTGCC | CTCCCCCTCCTCTGTTGCTG |
| **P-02** | 3 | 41266110 | TCACTGAGCTAACCCTGGCT | CCACTCAGAGAAGGAGCTGTGG |
| **P-05** | 3 | 12660100 | TGTGCTCCACAGGCAGATAA | CCGTGTTTGATGGCTCCAGC |
|  | 3 | 41266100 | TCACTGAGCTAACCCTGGCT | GCTGTGGTAGTGGCACCAGA |
|  | 11 | 100962605 | GCCAGCCTGACAGCACTTTC | CATTCAGCTGTGCCAGGCAG |
|  | 12 | 26749892 | AGGGATGCAGTCGTCATGGG | GGGGGTGAAGACGTGCTGAT |
|  | 22 | 36696277 | GCCAGCTTGCAGTTCTGGTC | GCGTTTGGATCTGGCCTTCG |
| **P-10** | 12 | 26568307 | AGGAGTTCGGTTTTTCAGCCT | ACACGAAATGGCCGCTCTAT |
| **P-11** | 11 | 532737 | GGAGAGCACACACTTGCAGC | GCAAGGCTTGATCCCACAGC |
| **P-13** | 1 | 11199401 | TACAGACCTTGCGGGCACTC | GAGTGACAACCACCCACCCA |
| **P-14** | 14 | 105236685 | CTATCGTCCAGCGCAGTCCA | GTGGGGCCCTGTCTCAGTTT |
| **P-16** | 12 | 26553126 | ACGAACACACAAAGAGTTGTAGT | AGGACGTGTGACACTCTCCT |
| **P-17** | 11 | 108117799 | GGGAGCTAGCAGTGTAAACAGA | AAGCCCAAAATGCCCAGTTT |
|  | 11 | 111625284 | CCCCATTCATTCCCTGTCAACC | TGGCCCTGTGAAAGCATTCT |
|  | 17 | 7578370 | GGAATCAGAGGCCTGGGGAC | ACAGTACTCCCCTGCCCTCA |
| **P-18** | 3 | 4709191 | TCACTGCCCTGCTCCACAAT | GAATGCACCCCCAGGTCTCA |
|  | 5 | 38962438 | TGTCAGGGCTAAGACCCACT | ATGTTTTTCTAGTGGCCAAATGT |
|  | 10 | 43610119 | AAGCCACCCATCTCCTCAGC | CCCTCACAGGATGGCCTCTG |
|  | 17 | 7577535 | AATCGGTAAGAGGTGGGCCC | TTGCCACAGGTCTCCCCAAG |
|  | 20 | 54961541 | GGGCTGCTTGCTCTTTTGGG | AGGCTACAGCTCCAGTTGGA |
| **P-21** | 3 | 41266124 | TGATGGAGTTGGACATGGCCA | GGACTGAGAAAATCCCTGTTCCC |
| **P-22** | 2 | 165997273 | GCAGGACGGTATGACAGCCT | AGACATGGAGAGCGACGCAA |
|  | 2 | 165997274 | GCAGGACGGTATGACAGCCT | AGACATGGAGAGCGACGCAA |
|  | 3 | 41266137 | TGGACATGGCCATGGAACCA | ACCAGCTACTTGTTCTTGAGTGA |
| **P-23** | 3 | 41266110 | TCACTGAGCTAACCCTGGCT | CAGAGAAGGAGCTGTGGTAGTG |
| **P-25** | 3 | 41268766 | GTCCAATGGCAAGCTGGCTG | CCAGCTTCTACAATAGCCGGC |
| **P-26** | 3 | 41266113 | TCACTGAGCTAACCCTGGCT | TGCCTTTACCACTCAGAGAAGGA |
|  | 4 | 55981463 | AGAGACGATTGGAGGAGATGCA | TGGTTGTGTGAGGTGTCCCT |
| **P-31** | 3 | 41266136 | TGATGGAGTTGGACATGGCCA | GGACTGAGAAAATCCCTGTTCCC |
|  | 6 | 44219910 | CTGTGTGCAGCAGCTCAAGG | CTTGTGAGGGAGCCCACCTC |
| **P-32** | 2 | 165948799 | CAGCTAGAAGGTCCTGGGGC | GGAAACGGATGACCAGGGCA |
|  | 3 | 41266101 | TCACTGAGCTAACCCTGGCT | GCTGTGGTAGTGGCACCAGA |
|  | 12 | 26636635 | TGGGTGGTGGTCATGACACA | ACTGAGTATTGCCAGGGCCC |
|  | 17 | 7577094 | GGGGAGAGGAGCTGGTGTTG | TTGAGGTGCGTGTTTGTGCC |
| **Hep-G2** | 4 | 55976843 | ATGGAATCTCTGGTGGAAGCC | GTGTGATTGGACTCAAGGGGTA |
|  | 10 | 43608351 | AGGATCTGCCTAGGAGGTGG | CACAGGAGGCTCAGCTTGAT |
|  | 19 | 18279692 | ATGGAGGACGAGGACGATCT | GTCCACTCACACCACGGAG |
| **SNU-449** | 1 | 156851421 | CACCAGAGGTCTACGCCATC | CCCACATGCTGAGGGTGAAT |
|  | 10 | 89717696 | TGTGGTCTGCCAGCTAAAGG | TGTCTCACCAATGCCAGAGT |
| **HUH-7** | 9 | 93606577 | GAGTCTGATGGCCTGGTCTG | CAGGAAGTCTGTGTGGGGTC |
|  | 12 | 18762561 | CAGACAAGAAGCCTAAGGTGC | TTGGCGGAGATACCCATTCT |
|  | 19 | 7141798 | AAATGACGTCACCAGCCCAA | AAGGGCCTTACCTCATCACC |
| **SNU-182** | 3 | 130409498 | TGTCCTGAGATCAGGGAGATTA | TCCAAGAAGCTGGACAGCAC |
|  | 13 | 29008268 | TGATCCCTGATGGAAAACGCA | ACAGAGCACTTCGGCTTATGT |
|  | 19 | 7184495 | TGCCTGGGCAACTGTTCTC | AGACCCACATCCAGAACTCACT |
| **SNU-423** | 3 | 130452809 | TTTTAATTCCTTTTCTTCCCTTGGT | CCAGCCCATTTTCCTTAGGC |
|  | 13 | 28913428 | TGAGTGTATGTGAATGTGGAGAAGT | TGCTGGCATCATAAGGGAGC |
| **SNU-475** | 13 | 28611336 | GCAATCATAAGCACCAGCCAG | ACACTTCAGCGTACAAAAACAG |
